# Osteoporosis, fracture and survival: Application of machine learning in breast cancer prediction models

**DOI:** 10.3389/fonc.2022.973307

**Published:** 2022-08-12

**Authors:** Lichen Ji, Wei Zhang, Xugang Zhong, Tingxiao Zhao, Xixi Sun, Senbo Zhu, Yu Tong, Junchao Luo, Youjia Xu, Di Yang, Yao Kang, Jin Wang, Qing Bi

**Affiliations:** ^1^ Department of Orthopedics, Zhejiang Provincial People’s Hospital, Hangzhou, China; ^2^ Department of Orthopedics, The Second Affiliated Hospital and Yuying Children's Hospital of Wenzhou Medical University, Wenzhou, China; ^3^ Department of Orthopedics, Hangzhou Medical College People`s Hospital, Hangzhou, China; ^4^ Center for Rehabilitation Medicine, Osteoporosis Center, Zhejiang Provincial People's Hospital (Affiliated People's Hospital, Hangzhou Medical College), Hangzhou, China; ^5^ Department of Orthopedics, Zhejiang Provincial People’s Hospital, Qingdao University, Qingdao, China; ^6^ Department of Psychology and Behavioral Sciences, Zhejiang University, Hangzhou, China; ^7^ Department of Orthopedics, The Second Affiliated Hospital of Soochow University, Suzhou, China; ^8^ Department of Musculoskeletal Oncology, Sun Yat-sen University Cancer Center, Guangzhou, China; ^9^ Department of Musculoskeletal Oncology, State Key laboratory of Oncology in South China, Guangzhou, China; ^10^ Department of Musculoskeletal Oncology, Collaborative Innovation Center for Cancer Medicine, Guangzhou, China

**Keywords:** breast cancer, estrogen, osteoporosis, fracture, prognosis, machine learning

## Abstract

The risk of osteoporosis in breast cancer patients is higher than that in healthy populations. The fracture and death rates increase after patients are diagnosed with osteoporosis. We aimed to develop machine learning-based models to predict the risk of osteoporosis as well as the relative fracture occurrence and prognosis. We selected 749 breast cancer patients from two independent Chinese centers and applied six different methods of machine learning to develop osteoporosis, fracture and survival risk assessment models. The performance of the models was compared with that of current models, such as FRAX, OSTA and TNM, by applying ROC, DCA curve analysis, and the calculation of accuracy and sensitivity in both internal and independent external cohorts. Three models were developed. The XGB model demonstrated the best discriminatory performance among the models. Internal and external validation revealed that the AUCs of the osteoporosis model were 0.86 and 0.87, compared with the FRAX model (0.84 and 0.72)/OSTA model (0.77 and 0.66), respectively. The fracture model had high AUCs in the internal and external cohorts of 0.93 and 0.92, which were higher than those of the FRAX model (0.89 and 0.86). The survival model was also assessed and showed high reliability *via* internal and external validation (AUC of 0.96 and 0.95), which was better than that of the TNM model (AUCs of 0.87 and 0.87). Our models offer a solid approach to help improve decision making.

## Introduction

Breast cancer is one of the most common malignancies in women worldwide and the leading cause of cancer-related death (1, 2). More than 2.3 million new cases and nearly 700,000 deaths from breast cancer are reported every year worldwide (3, 4). More than 250,000 people develop breast cancer in the United States each year, accounting for 30% of all cancers in women, and more than 40,000 people die from breast cancer, accounting for 14% of all cancer deaths (5). The survival rate of breast cancer patients is closely related to the economic status and economic conditions of the country and region (4), which impacts access to chemotherapeutic drugs, anti-estrogen treatment regimens and targeted drugs (6, 7).

Standard breast cancer treatment with chemotherapy, hormone therapy, or radiation therapy is associated with a good prognosis in breast cancer patients but may adversely affect bone tissue or lead to premature menopause and may increase the risk of osteoporosis (8, 9). Factors such as premature ovarian failure and premature menopause are associated with accelerated bone loss in premenopausal women who receive standard chemotherapy (10, 11). Headley et al. and Bruning et al. reported that, according to data provided by the National Cancer Database, an earlier age of menopause is observed in breast cancer patients treated with doxorubicin, cyclophosphamide, fluorouracil, and methotrexate in the previous 2 years, and this population has been found to have an increased risk of fracture at an earlier age than healthy populations (12–14). Not only is the treatment of breast cancer closely related to a decrease in bone mineral density, but the progression of breast cancer itself also aggravates bone mass loss. Breast cancer spreads easily to the bone. In fact, approximately 70% of advanced breast cancer patients develop bone metastases (15, 16). Notably, skeletal invasion by breast cancer cells is often associated with osteolytic lesions (17, 18), which leads to the formation of pathological osteoporosis. The migration of primary cancer to bone and subsequent metastatic behavior is regulated through the RANKL/RANK/OPG system (19–21). Patients with breast cancer who do not develop bone metastasis have been shown to have increased bone resorption. A study by Kanis et al. confirmed that the annual incidence of conical bone fractures in women who were followed up from their first breast cancer diagnosis was almost five times higher than that in the general population (22).

The Osteoporosis Self-Assessment Tool for Asians (OSTA) and Fracture Risk Assessment Tool (FRAX) are popular tools used in clinics to predict the risk of osteoporosis and fracture (23). We found that these assessment tools for osteoporosis and fractures often ignore some important factors, such as cancer treatment and laboratory indicators, and they lack sufficient specificity (24).

The purpose of this study was to establish a prediction model for osteoporosis in breast cancer patients as well as prediction models for fracture risk and survival rate incorporating osteoporosis. These models will provide clinicians with quantitative assessment of the risk of osteoporosis, fractures, and death in breast cancer patients during treatment.

## Materials and methods

### Patients

Patient data from 2012 to 2021 were obtained from the case record system of Zhejiang Provincial People’s Hospital and Second Affiliated Hospital of Soochow University, with an average follow-up of 52.19 months (Std=26.90)

**Figure 1:**
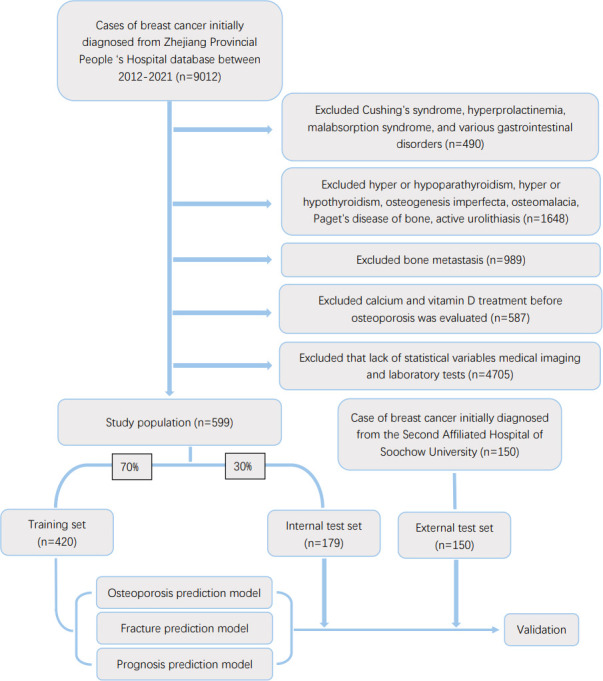
Flow diagram of the study population selected from Zhejiang Provincial People’s Hospital and the Second Affiliated Hospital of Soochou University. According to the inclusion and exclusion criteria, a total of 599 patient were included in this study,and they were randomly cut into the training and internal test sets in a 7:3 ratio. Data from the Second Affiliated Hospital of Soochou University as an external test set.

The inclusion criteria included primary breast cancer diagnosed by pathological examination. The exclusion criteria included the following: 1. the presence of underlying diseases causing secondary osteoporosis, such as Cushing’s syndrome, hyperprolactinemia, malabsorption syndrome and various gastrointestinal diseases (Crohn’s disease, chronic pancreatitis) 2. underlying diseases that affect bone metabolism, such as hyperparathyroidism or hypothyroidism, hyperthyroidism or hypothyroidism, osteogenesis imperfecta, osteomalacia, Paget’s disease of bone, or active urolithiasis; 3. patients who received osteoporosis prevention treatment before osteoporosis was evaluated, such as calcium and Vitamin D etc.; 4. the presence of bone metastases were found. 5. lack of information of accurate statistical variables (age, height, weight, smoking history, drinking history, family history, Karnofsky score), available imaging and laboratory tests (pathological examination of primary lesions, E-CT/PET-CT examination, blood biochemistry, reproductive hormones), available bone mineral density information from examination and whether received medication or special treatment (glucocorticoid use, chemotherapy drug use, targeted therapy drug use, radiation therapy, anti-estrogen drug use). We also performed a random phone survey of the patients to ensure data accuracy. The flow diagram of patient screening was mentioned in **Figure 1**.

The FRAX scores in this study were calculated by the FRAX official web version tool (25). The web address is https://www.sheffield.ac.uk/FRAX/. The FRAX score can be used to not only predict the risk of osteoporosis, but also assess the risk of fracture. Generally, the FRAX score used to predict hip fracture is labeled as FRAX-HF score.

Osteoporosis was diagnosed according to the 2022 AACE osteoporosis treatment guidelines (diagnostic criteria included 1. fragility fracture had occurred, whether or not less than -2.5; 2. the T value of osteopenia was >-2.5 and<=-1, and FRAX-HF score was > 3 or FRAX score was > 20; 3. the T value was less than -2.5) (26). The OSTA score was calculated by the following formula: OSTA= (body weight (kg) – age (years)) *0.2.

### Data collection

The variables selected for inclusion for osteoporosis predicting were as follows: age, BMI, smoking history, history of alcohol intake, M stage, molecular type of primary tumor, surgical treatment, anti-estrogen therapy, chemotherapy, targeted therapy, glucocorticoid medication, radiation therapy, family history of osteoporosis, fracture history, Karnofsky score less than 40, blood BALP level, blood calcium level, and blood phosphorus level. In the fracture prediction model, we added osteoporosis as a risk factor. In addition, we tested age, smoking history, history of alcohol intake, T and N stages, molecular type of primary tumor, surgical treatment, anti-estrogen therapy, chemotherapy, targeted therapy, glucocorticoid medication, radiation therapy, osteoporosis, brain metastasis, liver metastasis and lung metastasis as risk factors in the survival prediction model.

### Statistical analysis

All statistical analyses were performed using IBM SPSS Statistics (version 22), R software (version 4.1.0), and Python (version 3.8), and a P value<0.05 (two-sided) was considered statistically significant. All breast cancer patients were randomly divided into a training set and validation set. An independent t test was used to compare continuous data, and the chi-square test or Fisher’s exact test was used to compare categorical data. In the training cohort, univariate logistic analysis was performed to identify risk factors associated with osteoporosis. Variables with P< 0.05 in univariate analysis were incorporated into multivariate logistic regression to identify independent risk factors for osteoporosis in breast cancer patients. Correlation analysis was performed on the selected variables, and whether the variables affected each other was tested. Then, six different machine learning algorithms in Python, namely, decision tree (DT), random forest (RF), multilayer perceptron (MLP), logistic regression (LR), naive Bayes BS classifier (NBC) and eXtreme gradient boosting (XGB) were used to establish their own risk prediction models. In addition, the area under the receiver operating characteristic curve (AUC), accuracy score, sensitivity and specificity, correlation analysis heatmap, receiver operating characteristic (ROC) curve, and clinical decision curve analysis (DCA) were carried out to evaluate the performance of the models (27). Feature importance analysis was performed on the variables in the best machine learning model and was shown in the SHAP graph.

We calculated a number of different performance metrics in our analysis: accuracy, precision, sensitivity (recall), and F1-score. They are calculated to evaluate predictive capability with the number of true positives (TP), true negatives (TN), false positives (FP), and false negatives (FN) by the following equations: Accuracy indicates the overall correctness of the prediction:


$$\text{Accuracy}=\frac{\text{TP+TN}}{\text{TP+FN+TN+FN}}$$

Precision indicates the proportion of examples classified as positive that are actually positive:


$$\text{Precision =}\frac{\text{TP}}{\text{TP+FP}}$$

Sensitivity indicates the proportion of all positive examples that are paired, which measures the ability of the classifier to identify positive factors:


$$\text{Sensitivity}=\frac{\text{TP}}{\text{TP+FN}}=\text{recall}$$

The P and R indicators sometimes appear contradictory, so they need to be considered comprehensively. The most common method is the F-Measure (also known as the F-Score). The F-measure is the precision and recall weighted harmonic mean:


$$\text{F}=\frac{({\text{α}}^{2}+1)P*R}{{\text{α}}^{2}\left(P+R\right)}$$

When α=1, that is, F1 (28): $\text{F}1=\frac{2*P*R}{P+R}$

### Model visualization

We used web pages to establish risk assessment tools for osteoporosis, fractures, and survival in breast cancer patients. These tools can be used to visualize the machine learning models that achieve the best performance, and clinicians can directly log into the website to utilize the risk assessment tools.

## Results

By searching the case system of Zhejiang Provincial People’s Hospital, we found 9012 patients who were diagnosed with breast cancer from 2012 to 2021. According to rigorous screening, a total of 599 breast cancer patients were included as the target population to develop and internally validate the models. Of these patients, 114 (19%) patients developed osteoporosis, 41 (6.8%) patients experienced fractures during the tumor-bearing period, and 151 (25.2%) patients died during an average of 49.08 months (Std=26.93) of follow-up. Patients were randomly divided into training and validation groups (420 and 179 patients, respectively) to develop and validate the models. The randomness of the grouping was verified by the chi-square test and t-test, as shown in **Tables S1, S4** and **S7**.

The external validation cohort comprised 150 breast cancer patients from another independent center, of whom 28 patients (18.6%) developed osteoporosis, 12 patients (8%) experienced fractures during the tumor-bearing period, and 29 patients (19.3%) died during an average of 64.60 months (Std=22.98) of follow-up.

The distribution and characteristics of the osteoporosis, fracture, and survival variables are listed in **Tables S1, S3**, and **S5**. The age distribution of breast cancer patients with or without osteoporosis was as follows: median age: 63 years, range: 36-87 years (osteoporosis group); median age: 54 years, range: 30-92 years (nonosteoporosis group). The most common molecular type was HER+/HR+ (41.1%). Regarding treatment, almost all the patients underwent surgery (98%), nearly half of the training cohort did not receive anti-estrogen therapy (46.6%), 74 patients (17.6%) accepted targeted therapy, 218 patients (51.9%) received glucocorticoid therapy, and 106 patients (25.2%) were treated with radiotherapy. Moreover, the most common T and N stage were T1 (52.3%) and N0 (65.0%). In terms of distant metastasis of breast cancer to other organs, 7.8% of the patients had lung metastasis, 6.6% had liver metastasis, and 4.7% had brain metastasis.

In the external validation cohort, HER+/HR+ was the most common molecular type, accounting for 47.3% of the patients. A total of 141 (94%) patients received surgical treatment. More than half of the patients (57.4%) received different types of anti-estrogen therapy while 123 of them (82%) underwent chemotherapy. For other treatment, 32 patients (21.3%) received targeted therapy, 114 patients (76.6%) accepted glucocorticoid therapy, and 34 patients (22.6%) were treated with radiotherapy. In addition, we observed that lung metastasis account for the highest proportion among the three distant organ metastases (13 patients, 8.6%).

### Osteoporosis risk assessment model in breast cancer patients

Correlation tests were first performed among the different variables and the heatmap showed that almost of the variables were independent (**Figure S1**). The correlation coefficient between age and menopause was as high as 0.64. To ensure the rigor of the included variables, we retained the age variable and deleted menopause. Univariate logistic regression analysis identified multiple predisposing factors, including age, BMI, smoking history, alcohol drinking history, molecular type of primary tumor, M stage, anti-estrogen therapy, chemotherapy, targeted therapy, glucose corticosteroid medication, radiation therapy, family history of osteoporosis, fracture history, Karnofsky score below 40, BALP level, blood calcium level, and blood phosphorus level (P<0.05). These variables were selected for further multivariate logistic regression, which showed that age, BMI, molecular type of primary tumor, anti-estrogen therapy, chemotherapy, glucocorticoid medication, family history of osteoporosis, history of fracture, and blood BALP level were significantly correlated with the risk of osteoporosis in breast cancer patients (**Tables S3**).

We applied six different machine learning algorithms, namely, DT, RF, MLP, LR, NBC, and XGB, to establish osteoporosis risk prediction models, and compared the performance of the different models through ROC curve analysis of the training set as well as 5-fold cross-validation.

**Figure 2:**
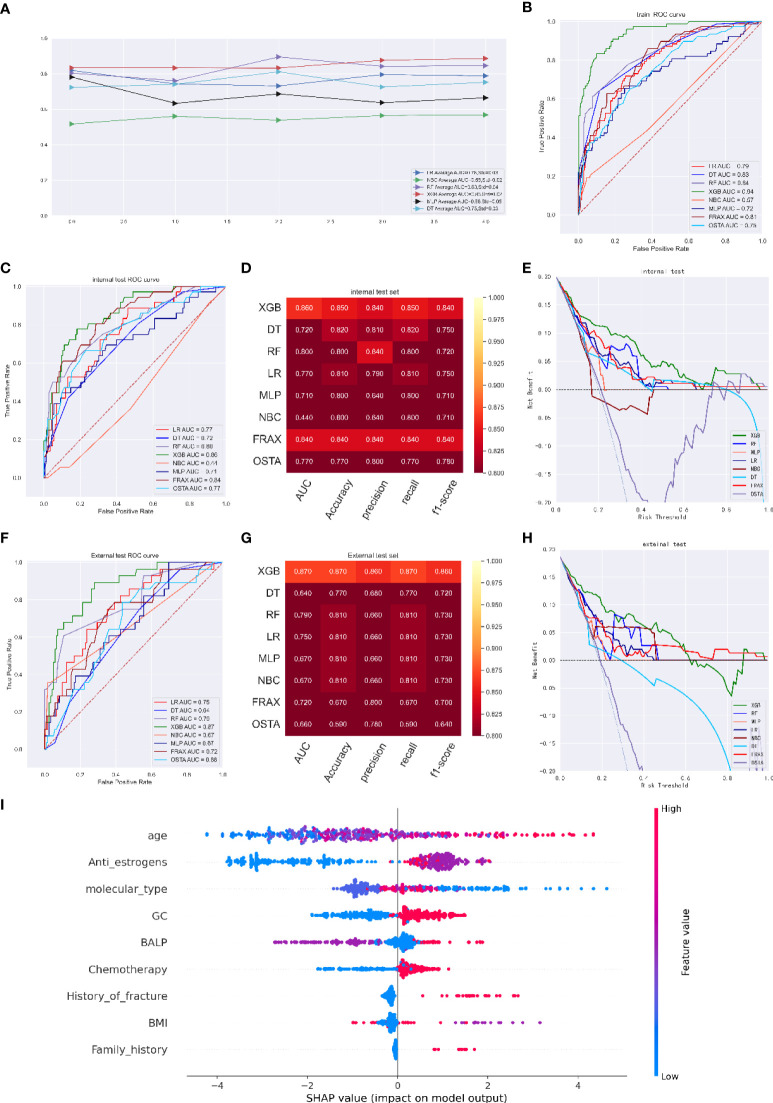
**(A)** Five-fold cross-validation results of different machine models in training set. Abbreviations: DT: Decision tree; LR: Logistic regression; MLP: Multilayer Pecepreon; NBC: Naive Bayes classification; RF: Random Forest; XGB: eXtreme gradient boosting. **(B)** The ROC curve of different machine learning models,FRAX score and OSTA score in training test set. **(C)** The ROC curve of different machine learning models, FRAX score and OSTA score in internal test set. **(D)** Prediction performance of different models, FRAX score and OSTA score in internal test set. **(E)** The DCA curve of different machine learning models, FRAX score and OSTA score in internal test set. **(F)** The ROC curve of different machine learning models, FRAX score and OSTA score in external test set. **(G)** Prediction performance of different models, FRAX score and OSTA score in external test set. **(H)** The DCA curve of different machine learning models, FRAX score and OSTA score in external test set. **(I)** Summary plots for SHAP values. For each feature, one point corresponds to a single patient. A point’s position along the x axis (i.e., the actual SHAP value) represents the impact that feature had on the model’s output for that specific patient. (osteoporosis predicting model).

As illustrated in **Figure 2A**, the XGB model exhibited better performance with an average AUC of 0.85 (Std=0.02) in five-fold cross-validation; the ROC curve showed an AUC of 0.94 (95%CI: 0.933 - 0.949) (**Figures 2A, B**). We defined an optimal cut-off probability of 0.50 for the XGB model so that in the internal validation set, the XGB model achieved the best AUC of 0.86 (accuracy of 0.85, precision of 0.84, sensitivity of 0.85, f1-score of 0.84), which was higher than that of FRAX and OSTA models (**Figures 2C, D**). We next evaluated the clinical utility of the model through DCA and achieved satisfactory results. We found that the clinical potency of the XGB model was dramatically higher than that of the FRAX tool, OSTA tool and other machine learning models (**Figure 2E**).

The independent validation cohort was used for external validation. The XGB model had an outstanding performance, with an AUC of 0.87, which was higher than the AUCs of the LR model (0.75), DT model (0.64), RF model (0.79), NBC model (0.67), MLP model (0.67), FRAX score model (0.72) and OSTA score model (0.66); the XGB model had an accuracy of 0.87, a precision of 0.86, a recall of 0.87, and an f1-score of 0.86 (**Figures 2F, G**). DCA indicated that the net benefit of the XGB model exceeded that of the FRAX score model, the OSTA score model and other models, indicating that it had better clinical impact at a wide range of threshold probabilities. (**Figure H**). Moreover, the prediction results of the models are presented as a heatmap in **Figure S2**.

SHAP values revealed the distribution of the impacts that each feature had on the XGB model for predicting osteoporosis in breast cancer patients (**Figure 2I**). Among them, age, anti-estrogen therapy, molecular type, glucocorticoid therapy and blood BALP level were the top five most predictive features in the model. Advanced age, treatment with anti-estrogen therapy and chemotherapy, the molecular type of cancer, glucocorticoids use, a higher BALP level, a lower BMI, a history of fracture, and a history of osteoporosis occurrence were associated with the risk of osteoporosis.

### Fracture risk assessment model in breast cancer patients

Fracture is the most common bone-related event that occurs in breast cancer patients. Except for fractures caused by bone metastasis, fracture evaluation for nonmetastatic causes such as osteoporosis is currently inadequate (29). To more comprehensively evaluate bone-related events in breast cancer patients, we next attempted to develop a fracture risk score model for breast cancer patients without bone metastasis.

Since the fracture risk factors are similar to those for osteoporosis, the variables mostly remained the same as those in the osteoporosis model. However, we added osteoporosis as a risk factor, which is missing in the current popular tool — the FRAX tool. Correlation test was performed first between the determined variables, as shown in **Figure S3**. The correlation coefficients of the included factors were all lower than 0.6 except age and menopause, confirming that there was no significant correlation between each factor after deleting menopause. None of the correlation scores between osteoporosis and other variables were above 0.26 (**Figure S3**).

After univariate and multivariate analyses, we found that age, BMI, chemotherapy, history of fracture, blood BALP level, and osteoporosis were independent risk factors for fracture in breast cancer patients. Details are shown in **Table S6**. In contrast to the FRAX tool, laboratory examination of the blood BALP level and history of osteoporosis played an important role in our model. Furthermore, we demonstrated that some variables such as smoking history, alcohol drinking history, and glucocorticoid medication use, which are included in the FRAX tool, did not meet the criteria for independent risk factors.

**Figure 3:**
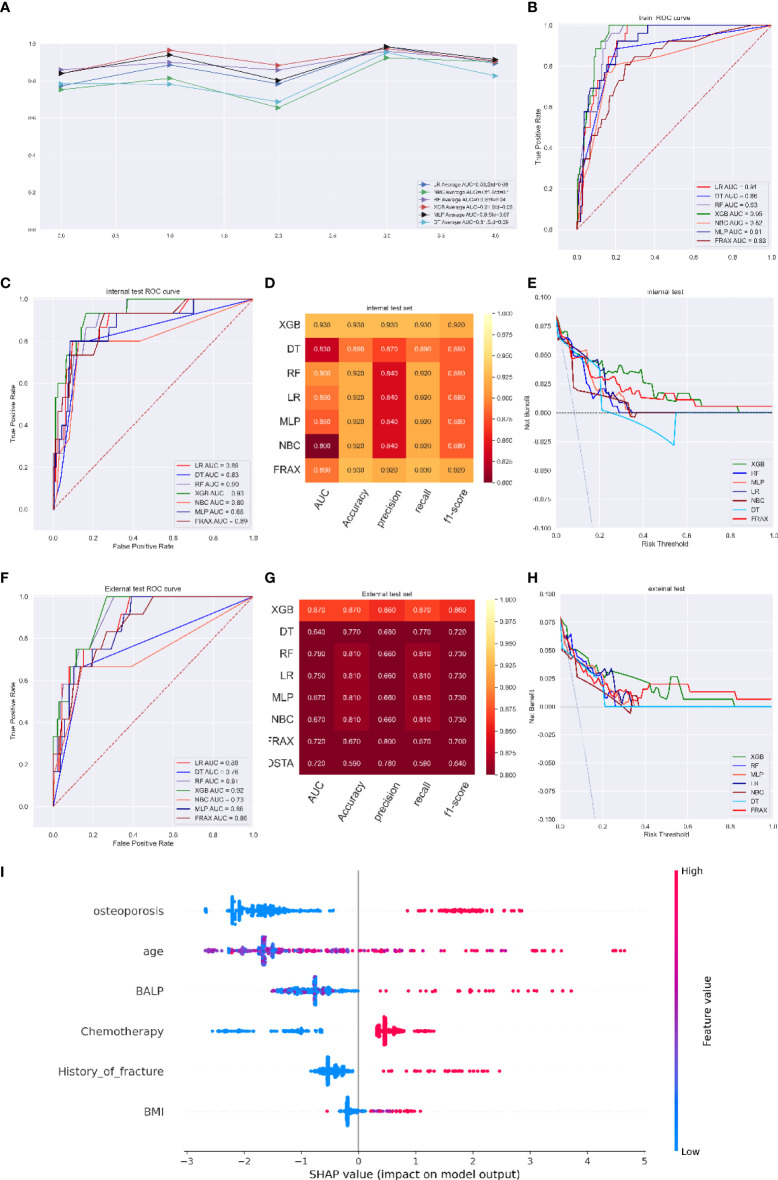
**(A)** Five-fold cross-validation results of different machine models in training set. **(B)** The ROC curve of different machine learning models, FRAX score in training test set. **(C)** The ROC curve of different machine learning models, FRAX score in internal test set. **(D)** Prediction performance of different models, FRAX score in internal test set. **(E)** The DCA curve of different machine learning models, FRAX score in internal test set. **(F)** The ROC curve of different machine learning models, FRAX score in external test set. **(G)** Prediction performance of different models, FRAX score in external test set. **(H)** The DCA curve of different machine learning models, FRAX score in external test set. **(I)** Feature importance plot for the XGB osteoporosis prediction model. All the features are shown in this figure. The blue and red points in each row represent nodules having low to high values of the specific feature, while the *x*-axis shows the SHAP value, indicating the impact on the model. (fracture predicting model).

In the fracture risk assessment model, the LR, NBC, RF, XGB, MLP, and DT models had average AUCs of 0.86 (Std=0.08), 0.81 (Std=0.10), 0.90 (Std=0.04), 0.91 (Std=0.05), 0.90 (Std=0.07), and 0.81 (Std=0.09), respectively. The XGB model performed better than any other model in ROC curve analysis and had an AUC of 0.93 (95%CI: 0.933-0.949) (**Figures 3A, B**). The optimal cut-off probability was set as 0.50 for the XGB model. In the internal test set, the XGB model achieved the best score, with an AUC of 0.93, an accuracy of 0.93, a precision of 0.93, a sensitivity of 0.93, and an f1-score of 0.90, while the FRAX score fracture model had an AUC of 0.89, an accuracy of 0.93, a precision of 0.92, a sensitivity of 0.93, and an f1-score of 0.92 (**Figures 3C, D**). We evaluated the clinical utility of the model through DCA. The curve representing the XGB model was considerably higher than the curves of the other models, which showed that the clinical potency of the XGB model was reliable (**Figure 3E**).

In the external validation set, although the DCA curve showed that the net benefit of the XGB model was similar to that of the FRAX model (**Figure 3H**), the superior performance of the XGB model in other estimate methods still existed, with an AUC of 0.92, an accuracy of 0.93, a precision of 0.91, a recall of 0.93, and an f1-score of 0.92 compared with that of the FRAX score model (AUC of 0.86, accuracy of 0.91, precision of 0.90, recall of 0.91, and f1-score of 0.91) (**Figures 3F, G**). The prediction results of the models are illustrated as a heatmap in **Figure S4**.

**Figure 3I** shows the top six variables with the highest SHAP values of the XGB model for predicting fracture. The most important factors associated with the predictive power of the model were osteoporosis, age, blood BALP level, chemotherapy, history of fracture, and BMI. Advanced age, a higher BALP value, a lower BMI, a diagnosis of osteoporosis, treatment with chemotherapy and a history of fracture were thought to increase the risk of bone metastasis free fracture.

### Survival risk assessment model in breast cancer patients

Osteoporosis is linked to increased mortality in breast cancer patients (30, 31). Thus, we further developed a survival model. The correlation test was used to assess the determined variables, as clarified in **Figure S7**. Among them, lung metastases, liver metastases, and brain metastases all had certain correlations with the N stage, with correlation scores of 0.382, 0.487 and 0.445, while the other variables had lower correlations after excluding menopause which had a high correlation coefficient with age, indicating that they were mutually independent. Univariate and multivariate Cox analyses showed that age, N stage, molecular type, chemotherapy, radiotherapy, osteoporosis, brain metastasis, liver metastasis, and lung metastasis were independent risk factors for survival in breast cancer patients. Details are shown in **Table S9**.

**Figure 4:**
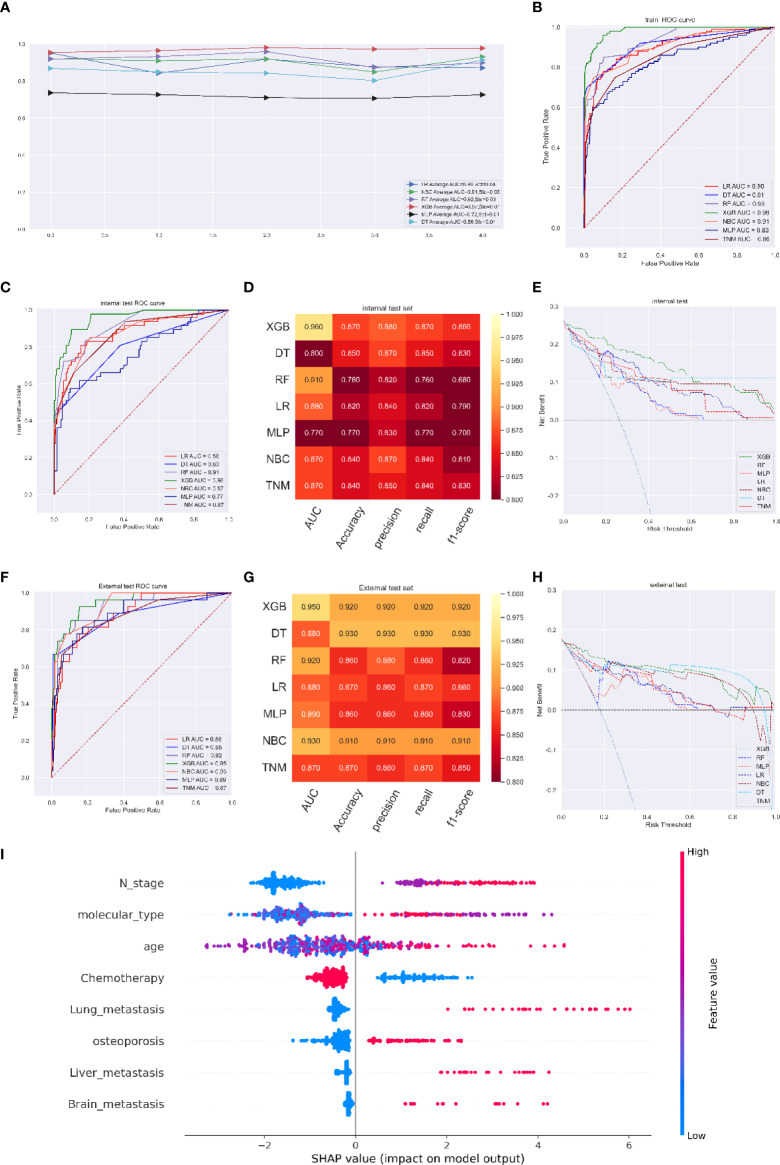
**(A)** Five-fold cross-validation results of different machine models in training set. **(B)** The ROC curve of different machine learning models, TNM stage model in training test set. **(C)** The ROC curve of different machine learning models and TNM stage model in internal test set. **(D)** Prediction performance of different models and TNM stage model in internal test set. **(E)** The DCA curve of different machine learning models and TNM stage model in internal test set. **(F)** The ROC curve of different machine learning models and TNM stage model in external test set. **(G)** Prediction performance of different models and TNM stage model in external test set. **(H)** The DCA curve of different machine learning models and TNM stage model in external test set. **(I)** Feature importance plot for the XGB osteoporosis prediction model. All the features are shown in this figure. The blue and red points in each row represent nodules having low to high values of the specific feature, while the *x*-axis shows the SHAP value, indicating the impact on the model. (survival predicting model for 8 years).

The XGB model had an excellent performance at the 3-year, 5-year and 8-year time points (average AUC of 0.93 at 3 years, std=0.03; 0.95 at 5 years, std=0.03; and 0.97 at 8 years, std=0.01). In ROC curve analysis, the XGB model had AUCs of 0.97 (95%CI: 0.9649-0.976) at 3 years, 0.99 (95%CI: 0.9868-0.9913) at 5 years, and 0.98 (95%CI: 0.9798-0.9858) at 8 years (**Figures 4A, B**). For the internal validation cohort, the XGB model had the best scores, with AUCs of 0.93 at 3 years, 0.93 at 5 years, and 0.96 at 8 years (**Figure 4C**). For the internal validation cohort, the XGB model had the best scores, with AUCs of 0.93 at 3 years, 0.93 at 5 years, and 0.96. The model performance evaluation indicators are shown in **Figures S9** and**4D**. DCA also verified that the XGB model achieved a higher titer in clinical treatment than any other model including the TNM stage model (**Figures 4E** and **S11**)

In the independent validation cohort of 150 patients, the ROC curve of the XGB model (AUC=0.96 at the 8-year time point) was superior to that of the current AJCC/TNM staging system (**Figure 4F**). In addition, the other parameters of the XGB model were an accuracy of 0.92, a precision of 0.92, a recall of 0.92, and an f1-score of 0.92, compared with those of the TNM staging system (accuracy of 0.87, precision of 0.86, recall of 0.87, f1-score of 0.85) (**Figure 4G**). Furthermore, the clinical benefit clarified by DCA of the XGB model was roughly as stable as that of the internal validation cohort and performed better than the TNM staging system (**Figure 4H**).

The SHAP summary plot of the predictive model ordered eight features based on their impact on the 8-year survival status. A lower SHAP value of a feature manifested a greater possibility of an 8-year survival. We found that a lower N stage, a lower age, and the application of chemotherapy were associated with higher possibility of 8-year survival, while the occurrence of osteoporosis, lung metastasis, liver metastasis and brain metastasis was associated with a lower possibility of 8-year survival (**Figure 4I**).

### Web predictor

**Figure 5:**
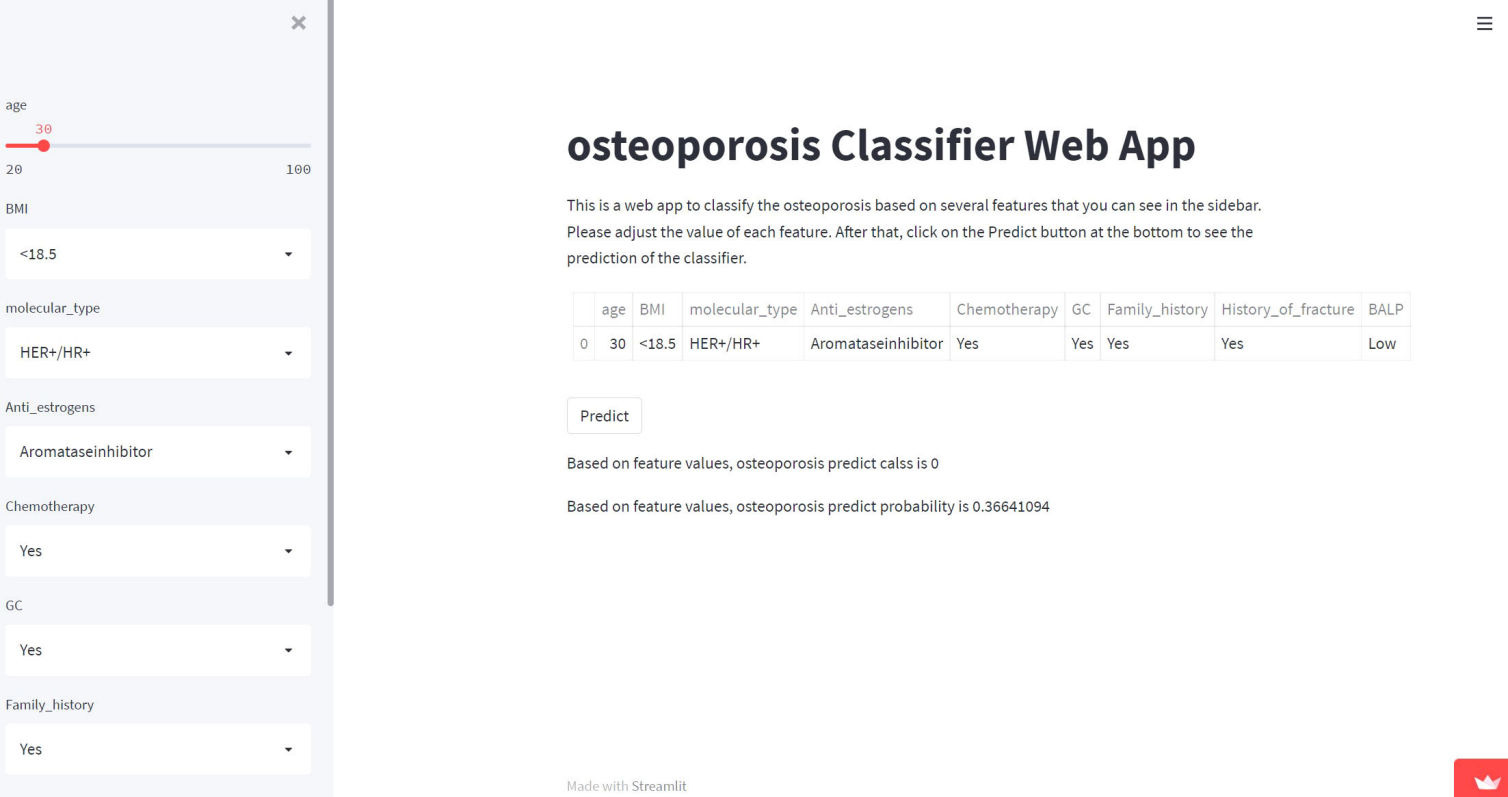
Screenshot of the web-based model. Screenshot of the XGB osteoporosis predicting model, which is available at https://share.streamlit.io/lry4000/osteoporosis/main.

A network predictor based on the best predictive performance of machine learning models was developed to predict osteoporosis, fracture occurrence and prognosis in breast cancer patients. The corresponding risk coefficient can be obtained by entering the variable through the sidebar of the webpage. (**Figure 5**)

Osteoporosis model: https://share.streamlit.io/lry4000/osteoporosis/main

Fracture model: https://share.streamlit.io/lry4000/fracture/main

Survival model:

3 Year https://share.streamlit.io/lry4000/survival_3/main

5 Year https://share.streamlit.io/lry4000/survival_5/main

8 Year https://share.streamlit.io/lry4000/survival_8/main

## Discussion

Osteoporosis is diagnosed in more than 7% of women with breast cancer each year, which is notably higher than the rate of 1-2% that is reported in postmenopausal women (24). Osteoporosis in breast cancer patients often leads to an increased risk of fracture (32, 33).

Chemotherapy is often used as adjuvant therapy for breast cancer patients (especially triple-negative breast cancer patients) to reduce the risk of recurrence after surgical resection (30, 34, 35). However, it often leads to premature menopause in women (36). Some studies have also shown that 97.4% of patients with locally advanced breast cancer who receive anthracycline-taxane neoadjuvant chemotherapy have vitamin D insufficiency or deficiency, which could increase the risk of osteoporosis through estrogen-independent mechanisms (24). In addition, anti-estrogen therapy is closely related to the occurrence of osteoporosis (37). Although the selective estrogen receptor modulator (SERM) tamoxifen has certain protective effects on bone, it is often less effective at preventing breast cancer recurrence. A study including 108 postmenopausal women showed that taking anastrozole (an aromatase inhibitor) for 5 years reduced lumbar spine and hip BMD by 6.08% and 7.24%, respectively, while taking tamoxifen for 5 years increased lumbar spine and hip BMD by 2.77 and 0.74%, respectively (38). Therefore, the use of aromatase inhibitors in the clinical treatment of osteoporosis deserves attention. In addition, glucocorticoids are widely used clinically to counteract the adverse reactions of chemical drugs, but large doses and long-term use of glucocorticoids often lead to osteoporosis. It is currently believed that glucocorticoids can lead to osteoporosis by promoting the differentiation and maturation of osteoclasts, inhibiting the generation of osteoblasts, and decreasing the production of insulin-like growth factor 1, growth hormones, etc. (39–41).

Surgery is the primary treatment for breast cancer. We searched the clinical information of breast cancer patients in the SEER database and found that the surgery rate is as high as 94.4%. This is roughly in line with the proportion of surgeries in our collected cases (98%). Such a predominantly high surgical rate reflects a general situation in breast cancer patients and the application scopes of our models in majority of the patients. More data is needed to assess whether our models perform similarly in patients with and without prior surgeries.

Patient mobility and exercise frequency often affect the occurrence of osteoporosis, but there is often a lack of quantitative standards. This model quantifies the patient’s exercise ability as the Karnofsky score to characterize the patient’s mobility, with 40 as the node, less than 40 as low mobility, and higher than 40 as high mobility, in line with the report that patients with low mobility are prone to osteoporosis (42, 43). Unfortunately, the Karnofsky score was not included in the osteoporosis model after regression analysis. Laboratory indicators such as blood calcium, blood phosphorus, and bone-specific alkaline phosphatase often reflect abnormal bone metabolism to a certain extent (44, 45). Through this analysis, we found that blood calcium and blood phosphorus did not meet the inclusion criteria, but the BALP level was absorbed in our model.

At present, according to clinical observations, neither the FRAX tool nor the OSTA tool can accurately predict the risk of osteoporosis and fractures in cancer patients. Breast cancer is currently the most common malignant tumor in the world. Clinicians lack reliable tools for predicting the risk of osteoporosis in breast cancer patients (29). In the process of breast cancer diagnosis and treatment, anti-osteoporosis interventions for people with a high risk of osteoporosis and fractures are ignored, so the opportunity to reduce the mortality rate and improve the quality of life of breast cancer patients prior to the osteoporosis stage is missed. Thus, it is difficult to prevent osteoporosis and fracture incidents in this population, which can lead to a substantial waste of social resources.

Machine learning has been increasingly applied in biomedicine in recent years to develop predictive models based on statistical associations between features in a given dataset. The learned model can then be used to predict any range of outputs, such as binary responses, categorical labels, or continuous values (46). In this study, we developed and validated the most commonly used machine learning algorithms to establish osteoporosis, fracture and survival models in breast cancer patients to predict the risk of related events. Through a comparison of different algorithms in multiple dimensions, we concluded that among these models, the model established by the XGB algorithm showed superior performance. The XGB algorithm uses a variety of methods to avoid overfitting, utilizes the second derivative of the loss function, supports and rowizes, and has faster processing speed (47). The osteoporosis and fracture model of breast cancer patients established by the XGB algorithm can provide doctors and patients with more accurate osteoporosis and fracture-related risks in clinical treatment and provide early intervention for high-risk groups. Through the application of zoledronic acid, denosumab or teriparatide and other anti-osteoporosis drugs, calcium supplementation, physical exercise, etc., can delay the occurrence of osteoporosis (48–50), and by weighing the treatment effect of breast cancer and the risk of osteoporosis, individualized treatment can be used to provide patients with a more appropriate treatment plan. In addition, we applied the survival assessment model for BC patients with osteoporosis risk factors to clinical practice, and the effect was better than that of the TNM staging model.

We constructed three models to predict osteoporosis, fractures and survival in breast cancer patients based on machine learning algorithms and dual-center data and developed a web-based predictor. Our models, both internally and externally validated, outperformed FRAX and OSTA, providing a new approach for screening high-risk populations of breast cancer with osteoporosis. After including the risk factors for osteoporosis and breast cancer-related factors, the performance of the fracture risk model was also distinctly better than that of the FRAX score model, and it provided a risk numerical reference for high-risk fracture groups. Meanwhile, the survival model, which included osteoporosis factors, also performed better than the TNM staging model.

?The model developed by machine learning in this project can allow clinicians to measure the possibility of osteoporosis in breast cancer patients after anti-cancer treatment and to follow up with anti-osteoporosis treatment (such as bisphosphonates and denosumab) to achieve the maximum clinical benefit. It is also possible to presume the incidence of osteoporosis, the possibility of fractures, and whether the survival rate after treatment can be improved before the standard treatment of breast cancer. This helps clinicians to decide whether to carry out related anti-cancer treatment (such as chemotherapy and anti-estrogen treatment) and whether to carry out fracture prevention.

## Data availability statement

The raw data supporting the conclusions of this article will be made available by the authors, without undue reservation.

## Ethics statement

This study was approved by Ethics Committee of Zhejiang Provincial People’s Hospital and the acceptance number was QT2022137.

## Author contributions

QB, YK and JW designed the project, reviewed and edited the manuscript. LJ, WZ wrote the manuscript. XZ, TZ and XS contributed to the literature retrieval. SZ, YT and JL carried out the research selection, data extraction and statistical analysis. YX and DY prepared the tables and figures. All authors contributed to this article and approved the submitted version.

## Funding

This study was supported by grants from National Science Foundation of China (Grant No. 81672769) and Major Science and Technology Projects of Zhejiang Province(2021C03078) as well as Medical Health Science and Technology Project of Zhejiang Provincial Health Commission, NO. 2022ky583.

## Acknowledgments

Thanks to Dr. Qifeng Ying for generously in providing us with osteoporosis-related data, and to others who participated in telephone contact and data entry.

## Conflict of interest

The authors declare that the research was conducted in the absence of any commercial or financial relationships that could be construed as a potential conflict of interest.

## Publisher’s note

All claims expressed in this article are solely those of the authors and do not necessarily represent those of their affiliated organizations, or those of the publisher, the editors and the reviewers. Any product that may be evaluated in this article, or claim that may be made by its manufacturer, is not guaranteed or endorsed by the publisher.
