# Supplementary material for: Osteoporosis, fracture and survival: Application of machine learning in breast cancer prediction models
Source: Front Oncol. 2022 Aug 12;12:973307. doi: 10.3389/fonc.2022.973307 (PMC9417646; doi:10.3389/fonc.2022.973307)
Supplement: Supplementary file 1 [file DataSheet_1.docx]

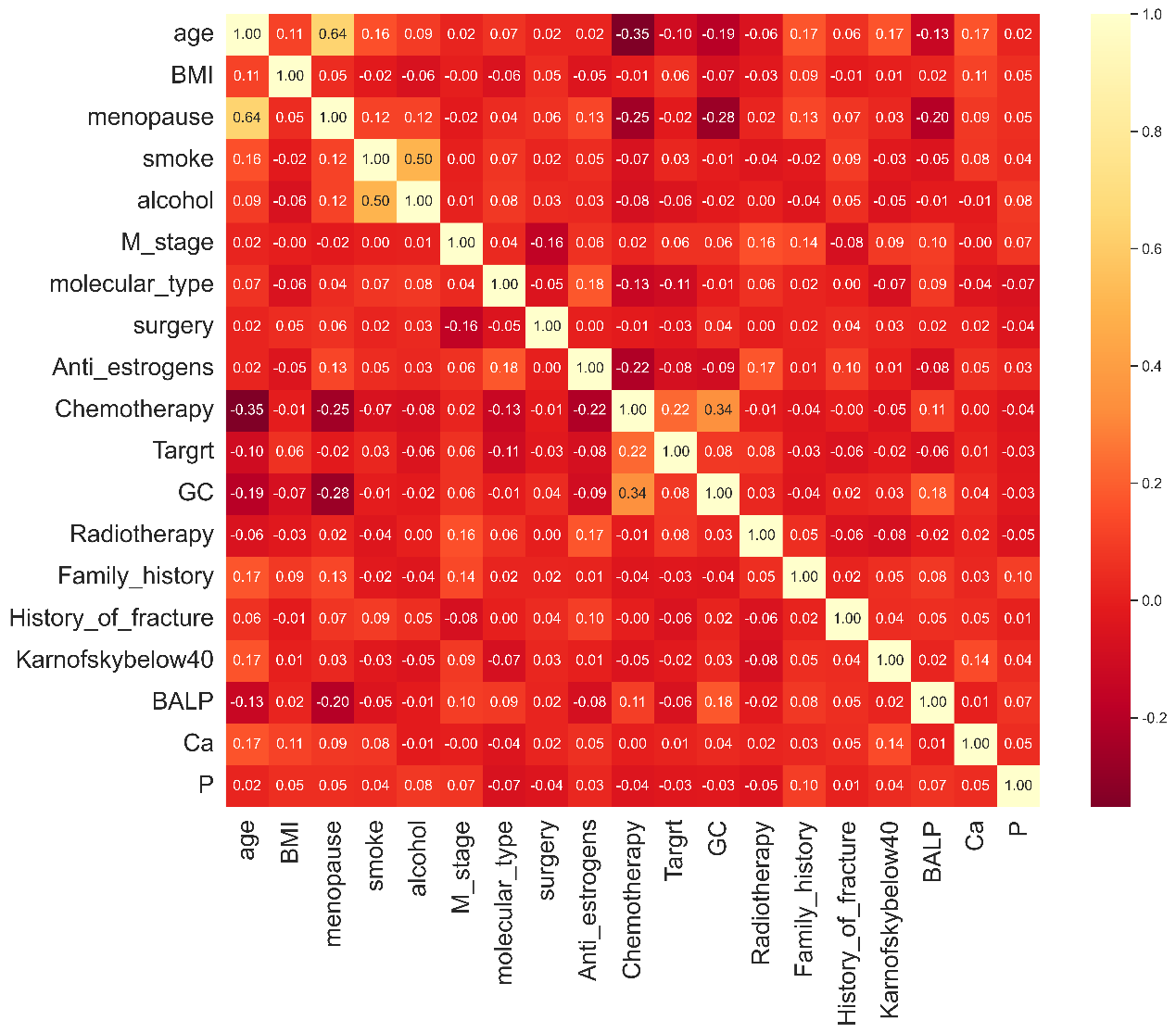


Figure S1. Results of correlation analysis between all variables of osteoporosis model. The heat map shows the correlation between the variables.


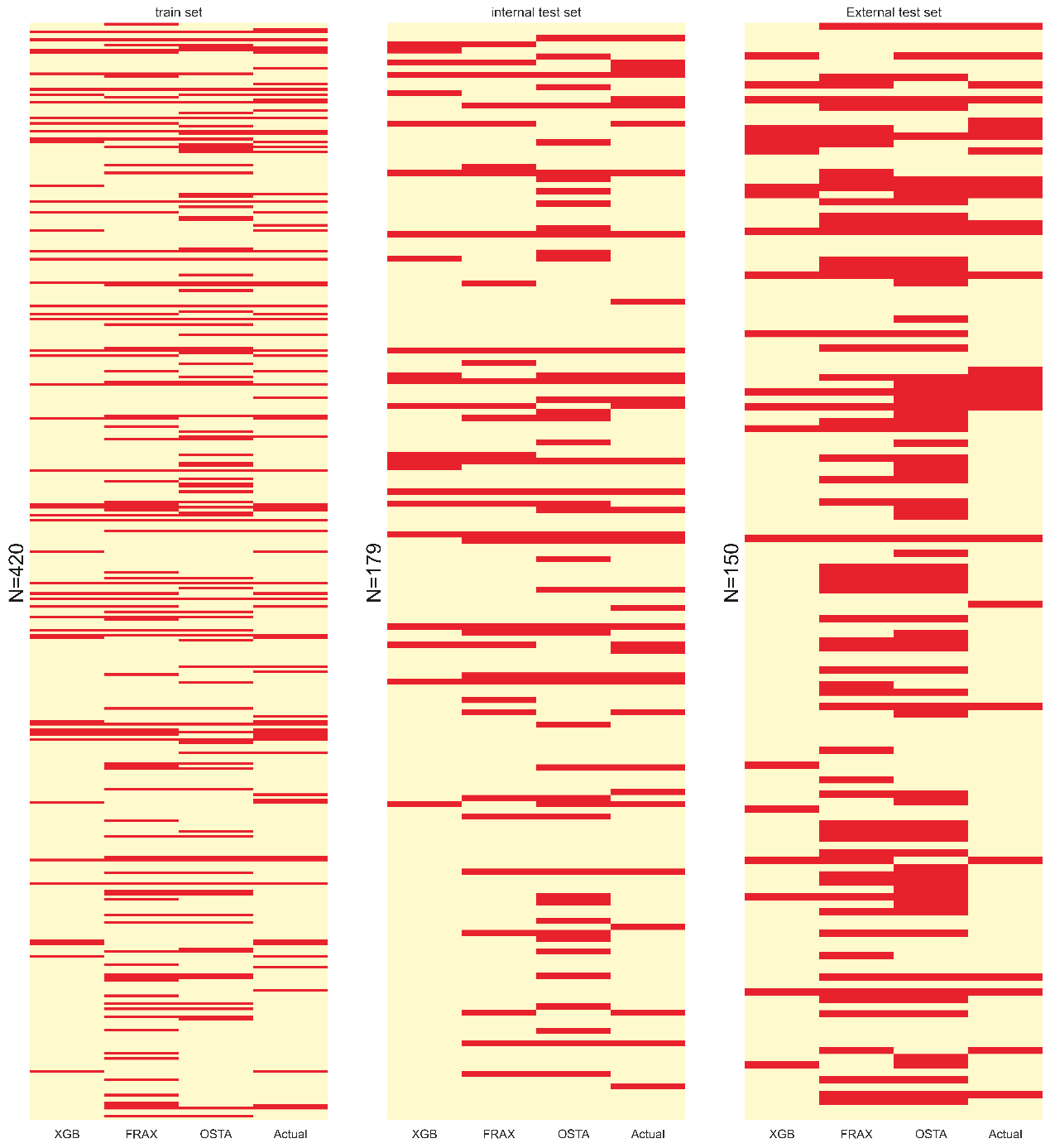


Figure S2. Prediction results of the XGB model and FRAX and OSTA score. The heat map shows the predicted results of XGB model versus the actual situation in internal test set and external test

in internal test set and external test set. Each column in the heat map represents the models’ predicted results of osteoporosis for all patients in the dataset. Dark colors represent bone osteoporosis cases and light colors are non-osteoporosis.


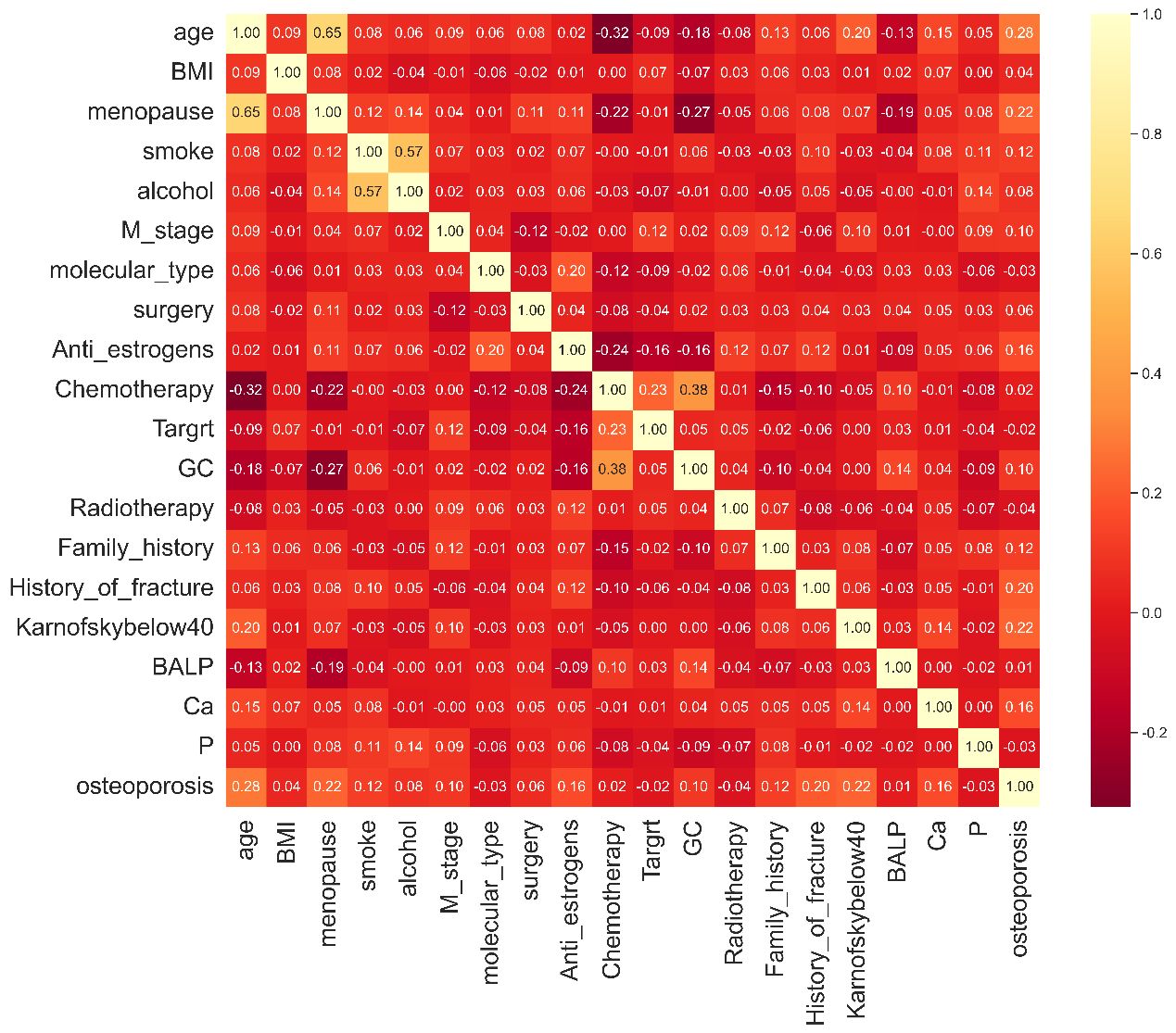


Figure S3. Results of correlation analysis between all variables of fracture model. The heat map shows the correlation between the variables


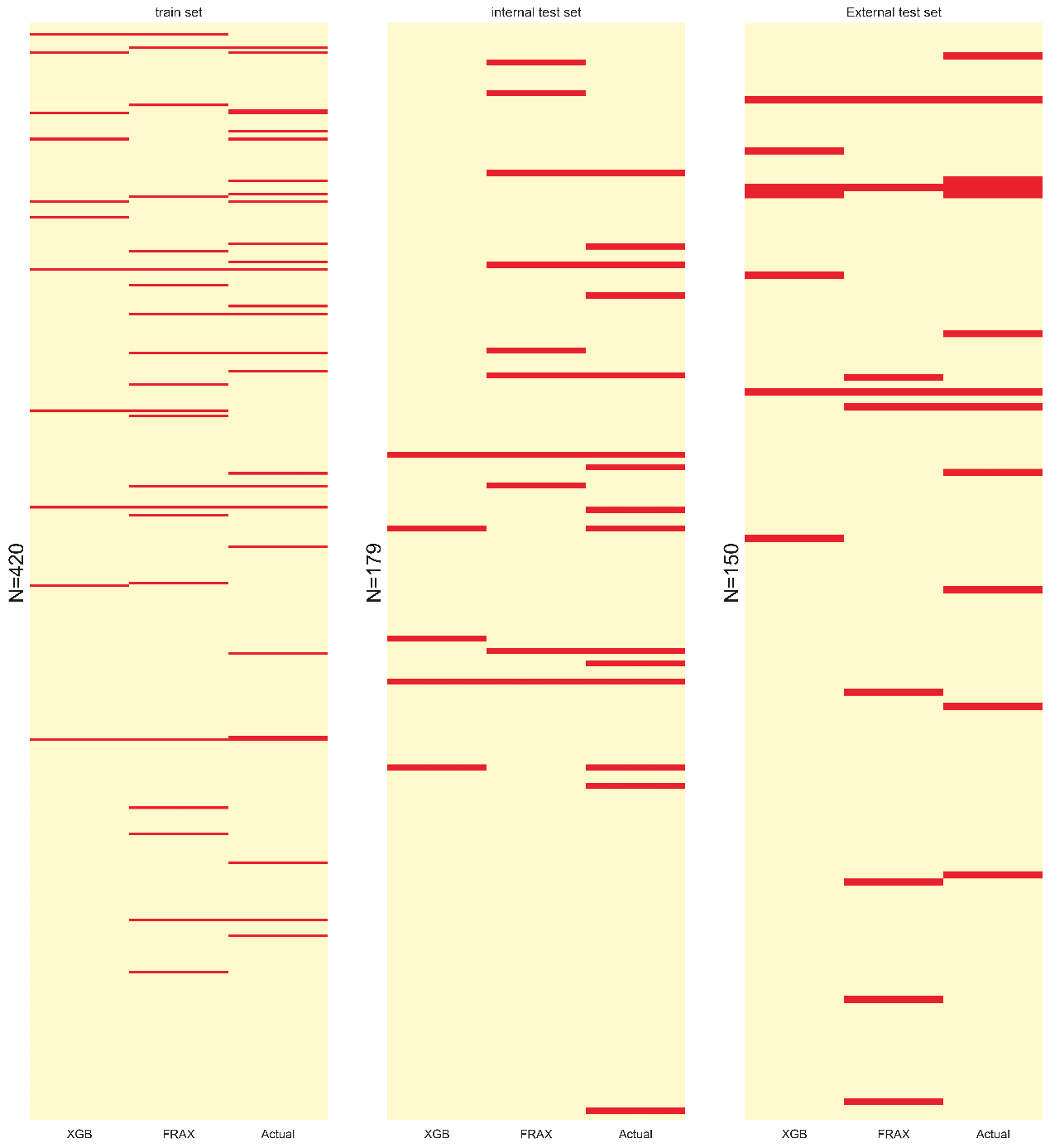


Figure S4. Prediction results of the XGB model and FRAX score. The heat map shows the predicted results of XGB model versus the actual situation in internal test set and external test


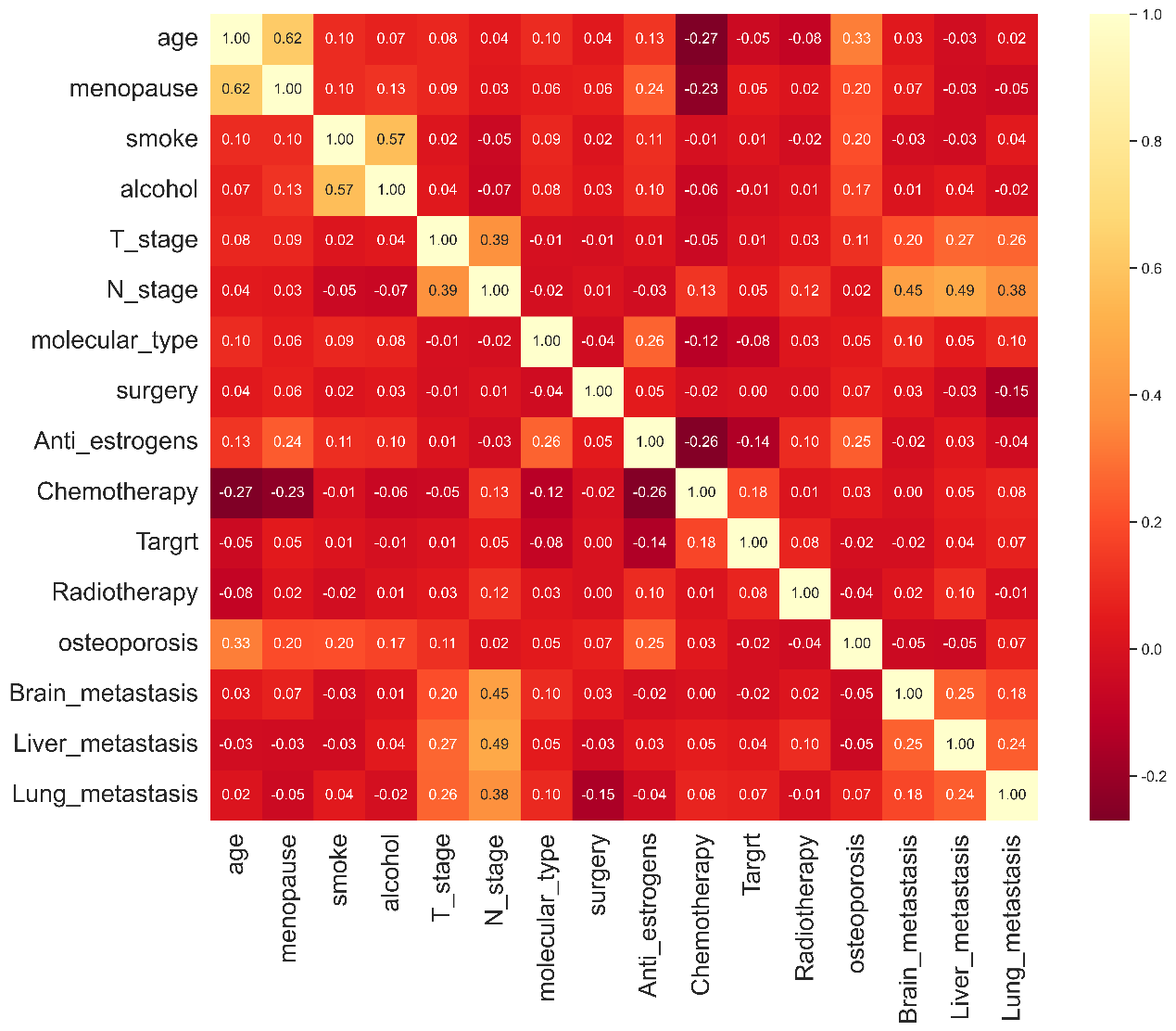


Figure S5. The heat map shows the correlation between the variables of predicting survive


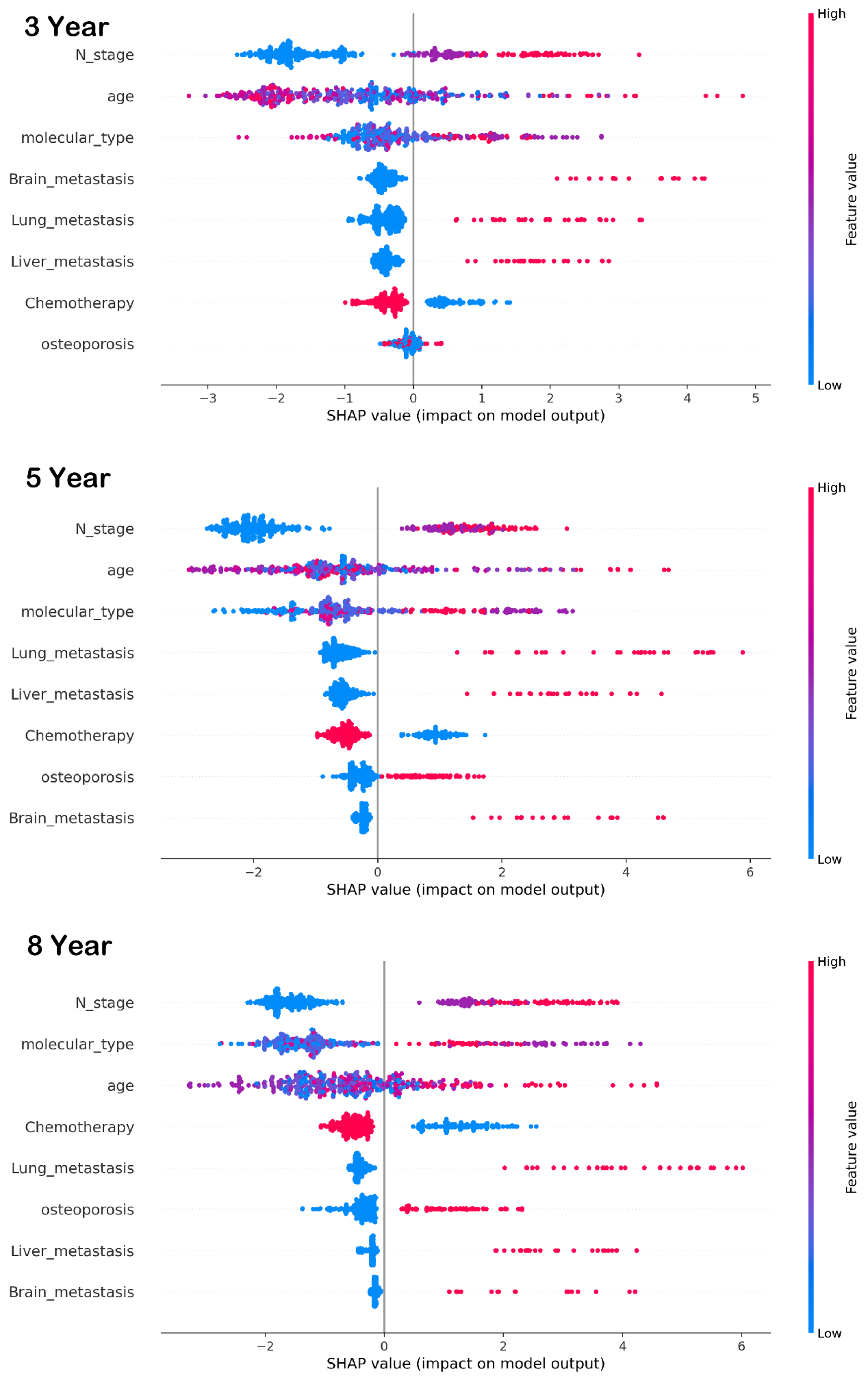


Figure S6. Feature importance plot for the XGB survival prediction model. All the features are shown in this figure. The blue and red points in each row represent nodules having low to high values of the specific feature, while the *x*-axis shows the SHAP value, indicating the impact on the model.


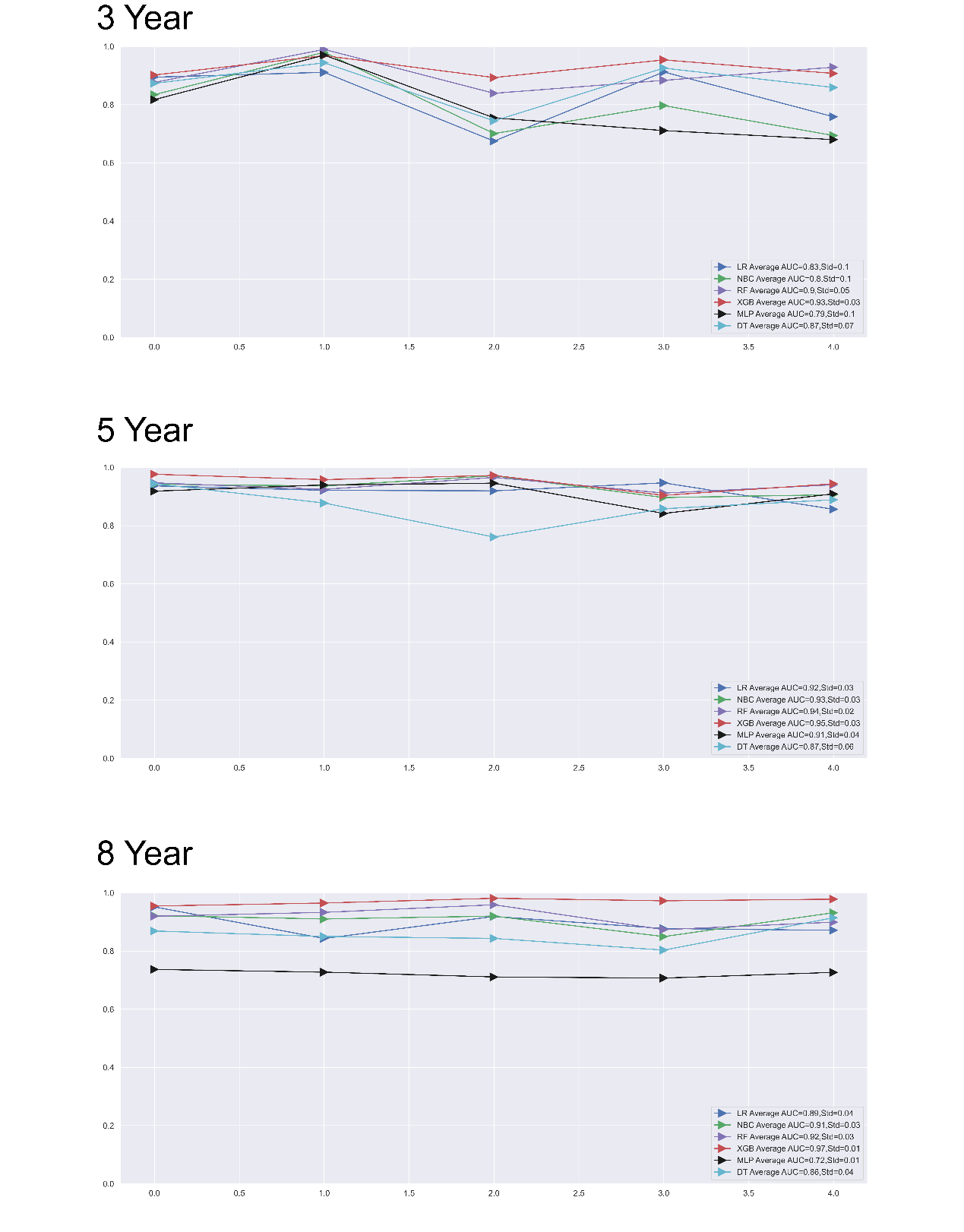


Figure S7. Five-fold cross-validation results of different machine models in training set. Abbreviations: DT: Decision tree ; LR: Logistic regression ; MLP: Multilayer Pecepreon ; NBC: Naive Bayes classification ; RF: Random Forest; XGB: eXtreme gradient boosting.


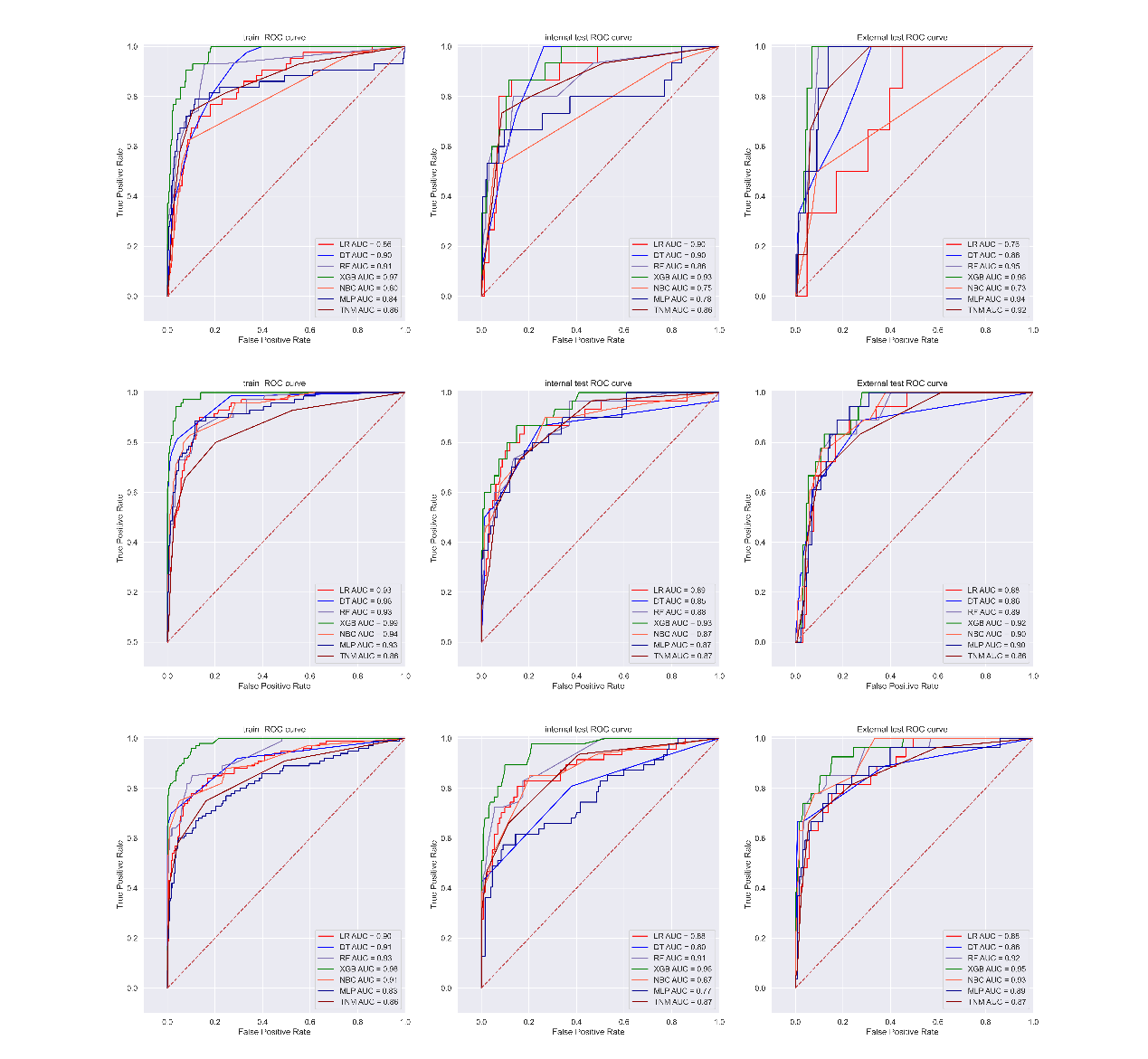


Figure S8. The ROC curve of different machine learning models and TNM stage model in training,internal and external test set.


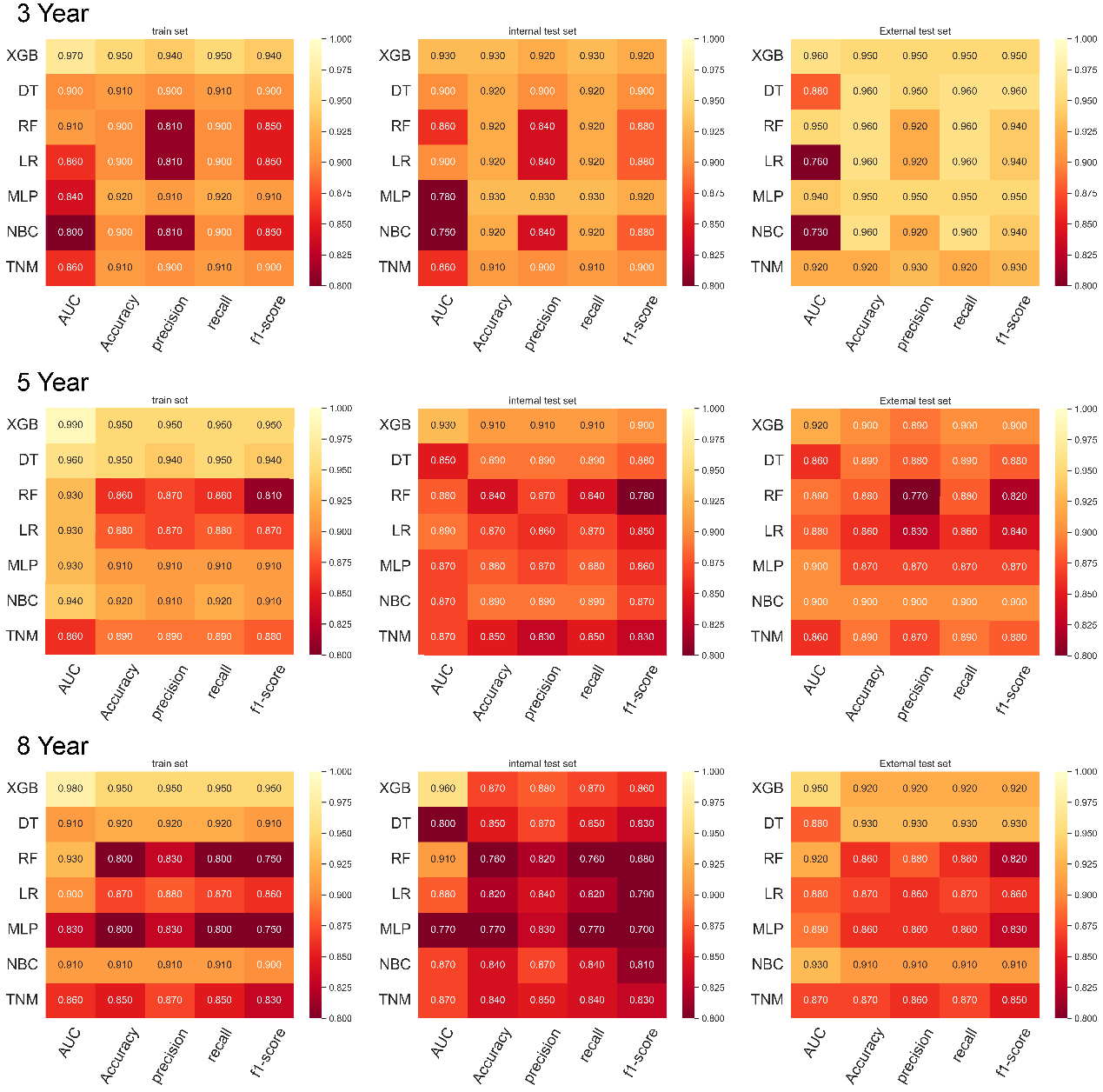


Figure S9. Prediction performance of different models,FRAX score ( survival model ).


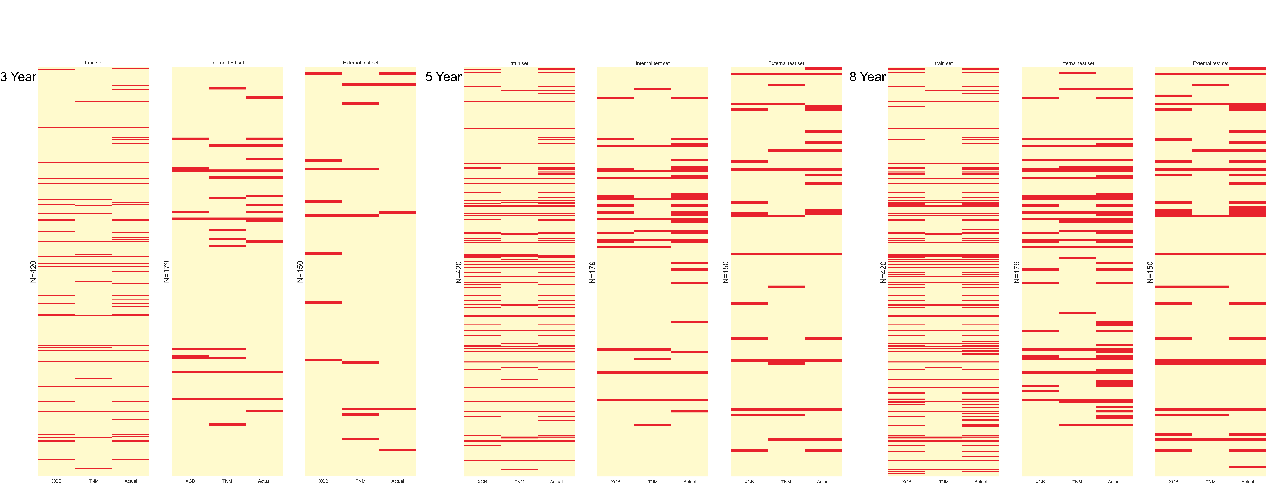


Figure S10. Prediction results of the XGB model and TNM model. The heat map shows the predicted results of XGB model versus the actual situation in internal test set and external test.


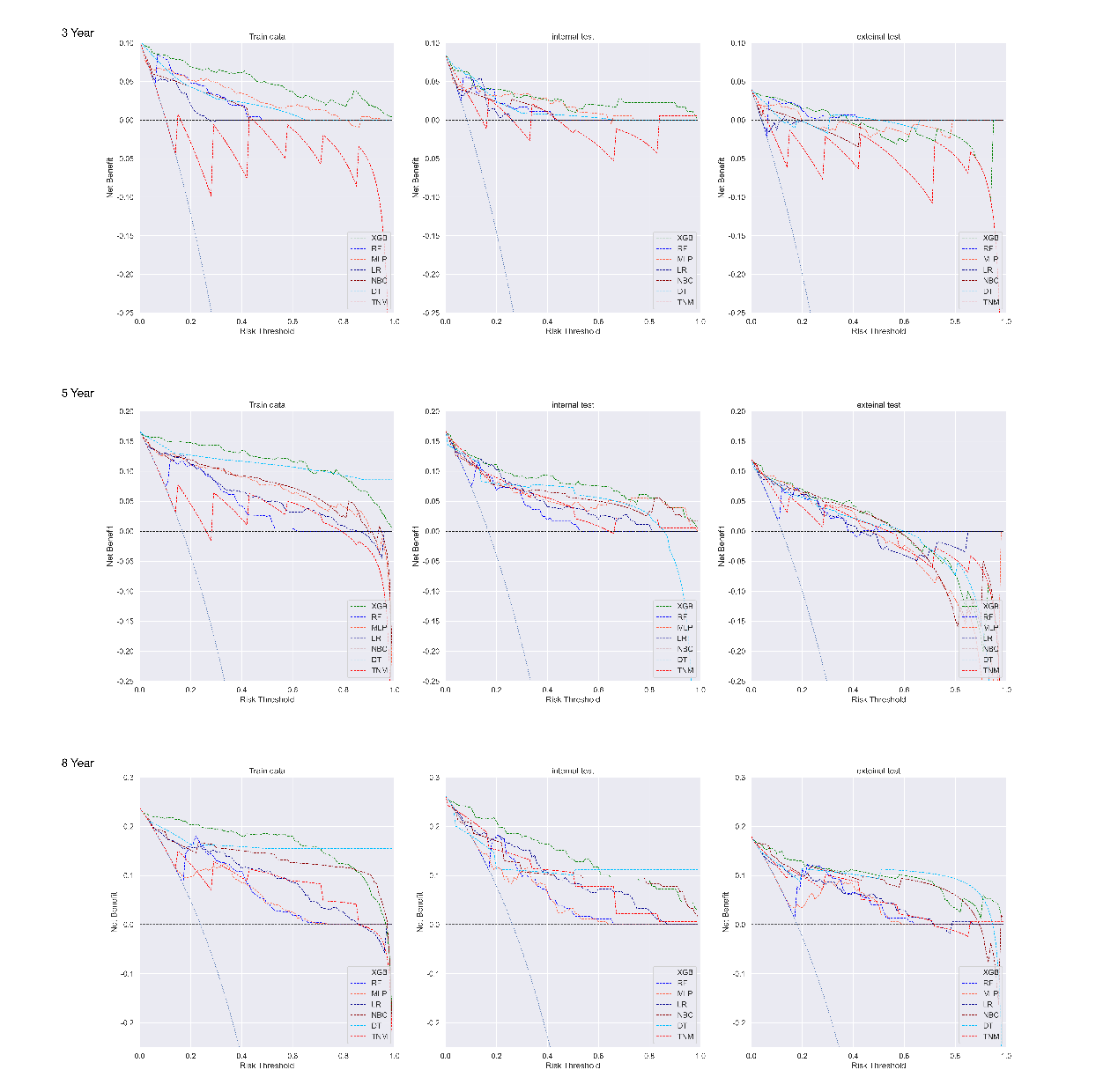


Figure S11. The DCA curve of different machine learning models ,TNM model in training,internal and external test set.
