# Supplementary material for: Osteoporosis, fracture and survival: Application of machine learning in breast cancer prediction models
Source: Front Oncol. 2022 Aug 12;12:973307. doi: 10.3389/fonc.2022.973307 (PMC9417646; doi:10.3389/fonc.2022.973307)
Supplement: Supplementary file 2 [file Table_1.docx]

|  |  |  |  |  |  |  |  |  |
| --- | --- | --- | --- | --- | --- | --- | --- | --- |
|  | **Training**  **group**  **(n=420)** | **%** | **Internal**  **Validation group**  **(n=179)** | **%** | **χ^2^** | **P** | **External validation group**  **(n=150)** | **%** |
| **BMI** |  |  |  |  | 3.480 | 0.176 |  |  |
| <18.5 | 16 | 3.8 | 10 | 5.5 |  |  | 2 | 1.3 |
| 18.5-27.99 | 373 | 88.8 | 149 | 83.2 |  |  | 142 | 94.6 |
| ≥28 | 31 | 7.3 | 20 | 11.1 |  |  | 6 | 4.0 |
| **Menopause** |  |  |  |  | 2.573 | 0.276 |  |  |
| No | 174 | 41.4 | 75 | 41.8 |  |  | 72 | 48.0 |
| Yes | 246 | 58.5 | 104 | 58.1 |  |  | 78 | 52.0 |
| **Smoke** |  |  |  |  | 2.643 | 0.267 |  |  |
| No | 412 | 98.0 | 176 | 98.3 |  |  | 147 | 98.0 |
| Yes | 8 | 1.9 | 3 | 1.6 |  |  | 3 | 2.0 |
| **Alcohol** |  |  |  |  | 3.301 | 0.192 |  |  |
| No | 397 | 94.5 | 174 | 97.2 |  |  | 145 | 96.6 |
| Yes | 23 | 5.4 | 5 | 2.7 |  |  | 5 | 3.3 |
| **M stage** |  |  |  |  | 2.215 | 0.330 |  |  |
| 0 | 370 | 88.0 | 151 | 84.3 |  |  | 128 | 85.3 |
| 1 | 50 | 11.9 | 28 | 15.6 |  |  | 22 | 14.6 |
| **Molecular type** |  |  |  |  | 3.944 | 0.414 |  |  |
| HER+/HR- | 105 | 25.0 | 36 | 20.1 |  |  | 22 | 14.6 |
| HER+/HR+ | 173 | 41.1 | 72 | 40.2 |  |  | 71 | 47.3 |
| Triple-negtive | 43 | 10.2 | 19 | 10.6 |  |  | 14 | 9.3 |
| LuminalA | 53 | 12.6 | 25 | 13.9 |  |  | 28 | 18.6 |
| LuminalB | 46 | 10.9 | 27 | 15.0 |  |  | 15 | 10.0 |
| **Surgery** |  |  |  |  | 2.479 | 0.290 |  |  |
| No | 8 | 1.9 | 2 | 1.1 |  |  | 9 | 6.0 |
| Yes | 412 | 98 | 177 | 98.8 |  |  | 141 | 94.0 |
| **Anti-estrogens** |  |  |  |  | 1.197 | 0.730 |  |  |
| No | 196 | 46.6 | 78 | 43.5 |  |  | 64 | 42.6 |
| Aromataseinhibitor | 183 | 43.5 | 86 | 48 |  |  | 54 | 36.0 |
| Anti-estrogenR | 35 | 8.3 | 12 | 6.7 |  |  | 30 | 20.0 |
| Combination | 6 | 1.4 | 3 | 1.6 |  |  | 2 | 1.3 |
| **Chemotherapy** |  |  |  |  | 3.687 | 0.158 |  |  |
| No | 120 | 28.5 | 57 | 31.8 |  |  | 27 | 18.0 |
| Yes | 300 | 71.4 | 122 | 68.1 |  |  | 123 | 82.0 |
| **Targeted therapy** |  |  |  |  | 4.552 | 0.103 |  |  |
| No | 346 | 82.3 | 158 | 88.2 |  |  | 118 | 78.6 |
| Yes | 74 | 17.6 | 21 | 11.7 |  |  | 32 | 21.3 |
| **GC** |  |  |  |  | 2.578 | 0.276 |  |  |
| No | 202 | 48.0 | 87 | 48.6 |  |  | 36 | 24.0 |
| Yes | 218 | 51.9 | 92 | 51.3 |  |  | 114 | 76.0 |
| **Radiotherapy** |  |  |  |  | 2.294 | 0.232 |  |  |
| No | 314 | 74.7 | 137 | 76.5 |  |  | 116 | 77.3 |
| Yes | 106 | 25.2 | 42 | 23.4 |  |  | 34 | 22.6 |
| **Family history** |  |  |  |  | 2.214 | 0.331 |  |  |
| No | 410 | 97.6 | 168 | 93.8 |  |  | 145 | 96.6 |
| Yes | 10 | 2.3 | 11 | 6.1 |  |  | 5 | 3.3 |
| **History of fracture** |  |  |  |  | 2.333 | 0.311 |  |  |
| No | 389 | 92.6 | 163 | 91.0 |  |  | 121 | 80.6 |
| Yes | 31 | 7.3 | 16 | 8.9 |  |  | 29 | 19.3 |
| **Karnofskybelow40** |  |  |  |  | 2.294 | 0.318 |  |  |
| No | 405 | 96.4 | 169 | 94.4 |  |  | 144 | 96.0 |
| Yes | 15 | 3.5 | 10 | 5.5 |  |  | 6 | 4.0 |
| **BALP** |  |  |  |  | 5.94 | 0.051 |  |  |
| Low | 128 | 30.4 | 40 | 22.3 |  |  | 12 | 8.0 |
| Medium | 258 | 61.4 | 120 | 67.0 |  |  | 126 | 84.0 |
| High | 34 | 8.0 | 19 | 10.6 |  |  | 12 | 8.0 |
| **Ca** |  |  |  |  | 2.247 | 0.325 |  |  |
| Low | 22 | 5.2 | 9 | 5.0 |  |  | 18 | 12.0 |
| Medium | 367 | 87.3 | 155 | 86.5 |  |  | 119 | 79.3 |
| High | 31 | 7.3 | 15 | 8.3 |  |  | 13 | 8.6 |
| **P** |  |  |  |  | 0.398 | 0.819 |  |  |
| Low | 9 | 2.1 | 7 | 3.9 |  |  | 7 | 4.6 |
| Medium | 392 | 93.3 | 169 | 94.4 |  |  | 129 | 86.0 |
| High | 19 | 4.5 | 3 | 1.6 |  |  | 14 | 9.3 |

Table S1.Baseline clinical characteristics of breast cancer patients in osteoporosis model.( We used t-test to verify the randomness of the age factor, p=0.597 (95%CI, -1.94-1.95))

|  | **Training Cohort** | | | | **Internal Validation Cohort** | | | **External Validation Cohort** | | | | |
| --- | --- | --- | --- | --- | --- | --- | --- | --- | --- | --- | --- | --- |
|  | **Osteoporosis** | **Normal** | **Total** |  | **Osteoporosis** | **Normal** | **Total** | **Osteoporosis** | | **Normal** | | **Total** |
| **Variable** | N=78 | N=342 | N=420 | P | N=36 | N=143 | N=179 | N=20 | N=122 | | N=150 | |
| **Age** | - | - | - | - | - | - | - | - | - | | - | |
| BMI |  |  |  | <0.001 |  |  |  |  |  | |  | |
| <18.5 | 9 | 7 | 16 |  | 7 | 3 | 10 | 1 | 1 | | 2 | |
| 18.5-27.99 | 65 | 308 | 373 |  | 26 | 123 | 149 | 27 | 115 | | 142 | |
| ≥28 | 4 | 27 | 31 |  | 3 | 17 | 20 | 0 | 6 | | 6 | |
| **Menopause** |  |  |  | <0.001 |  |  |  |  |  | |  | |
| No | 16 | 158 | 174 |  | 4 | 71 | 75 | 5 | 67 | | 72 | |
| Yes | 62 | 184 | 296 |  | 32 | 72 | 104 | 23 | 55 | | 78 | |
| **Smoke** |  |  |  | <0.001 |  |  |  |  |  | |  | |
| No | 71 | 341 | 412 |  | 36 | 140 | 176 | 28 | 119 | | 147 | |
| Yes | 7 | 1 | 8 |  | 0 | 3 | 3 | 0 | 3 | | 3 | |
| **Alcohol** |  |  |  | <0.001 |  |  |  |  |  | |  | |
| No | 67 | 330 | 397 |  | 35 | 139 | 174 | 28 | 119 | | 147 | |
| Yes | 11 | 12 | 23 |  | 1 | 4 | 5 | 0 | 5 | | 5 | |
| **M stage** |  |  |  | 0.090 |  |  |  |  |  | |  | |
| 0 | 65 | 305 | 370 |  | 30 | 121 | 151 | 23 | 105 | | 128 | |
| 1 | 14 | 37 | 50 |  | 6 | 22 | 28 | 5 | 17 | | 22 | |
| **Molecular type** |  |  |  | 0.918 |  |  |  |  |  | |  | |
| HER+/HR- | 18 | 87 | 105 |  | 5 | 31 | 36 | 3 | 19 | | 22 | |
| HER+/HR+ | 33 | 140 | 173 |  | 19 | 53 | 72 | 10 | 61 | | 71 | |
| Triple-negtive | 7 | 36 | 43 |  | 3 | 16 | 19 | 5 | 9 | | 14 | |
| LuminalA | 12 | 41 | 53 |  | 4 | 21 | 25 | 6 | 22 | | 28 | |
| LuminalB | 8 | 38 | 46 |  | 5 | 22 | 27 | 4 | 11 | | 35 | |
| **Surgery** |  |  |  | 0.361 |  |  |  |  |  | |  | |
| No | 0 | 8 | 8 |  | 0 | 2 | 2 | 1 | 8 | | 9 | |
| Yes | 78 | 334 | 412 |  | 36 | 141 | 177 | 27 | 114 | | 141 | |
| **Anti-estrogens** |  |  |  | <0.001 |  |  |  |  |  | |  | |
| No | 13 | 183 | 196 |  | 8 | 70 | 78 | 7 | 57 | | 64 | |
| Aromataseinhibitor | 60 | 123 | 183 |  | 27 | 59 | 86 | 20 | 34 | | 54 | |
| Anti-estrogenR | 3 | 32 | 35 |  | 1 | 11 | 12 | 1 | 29 | | 30 | |
| Combination | 2 | 4 | 6 |  | 0 | 3 | 3 | 0 | 2 | | 2 | |
| **Chemotherapy** |  |  |  | 0.721 |  |  |  |  |  | |  | |
| No | 21 | 99 | 120 |  | 13 | 44 | 57 | 3 | 24 | | 27 | |
| Yes | 57 | 243 | 300 |  | 23 | 99 | 122 | 25 | 98 | | 123 | |
| **Targeted therapy** |  |  |  | 0.823 |  |  |  |  |  | |  | |
| No | 65 | 283 | 346 |  | 32 | 126 | 158 | 20 | 98 | | 118 | |
| Yes | 13 | 61 | 74 |  | 4 | 17 | 21 | 8 | 24 | | 32 | |
| **GC** |  |  |  | 0.032 |  |  |  |  |  | |  | |
| No | 29 | 173 | 202 |  | 11 | 76 | 87 | 4 | 32 | | 36 | |
| Yes | 13 | 61 | 74 |  | 25 | 67 | 92 | 24 | 90 | | 114 | |
| **Radiotherapy** |  |  |  | 0.843 |  |  |  |  |  | |  | |
| No | 59 | 255 | 314 |  | 29 | 108 | 137 | 22 | 94 | | 116 | |
| Yes | 19 | 87 | 106 |  | 7 | 35 | 42 | 6 | 28 | | 34 | |
| **Family history** |  |  |  | <0.001 |  |  |  |  |  | |  | |
| No | 70 | 340 | 410 |  | 33 | 135 | 168 | 23 | 122 | | 145 | |
| Yes | 8 | 2 | 10 |  | 3 | 88 | 11 | 5 | 0 | | 55 | |
| **History of fracture** |  |  |  | <0.001 |  |  |  |  |  | |  | |
| No | 62 | 327 | 389 |  | 27 | 136 | 163 | 11 | 110 | | 121 | |
| Yes | 16 | 15 | 31 |  | 9 | 7 | 16 | 17 | 12 | | 29 | |
| **Karnofsky below40** |  |  |  | 0.004 |  |  |  |  |  | |  | |
| No | 71 | 334 | 405 |  | 29 | 140 | 169 | 27 | 117 | | 144 | |
| Yes | 7 | 8 | 15 |  | 7 | 3 | 10 | 1 | 5 | | 6 | |
| **BALP** |  |  |  | <0.001 |  |  |  |  |  | |  | |
| Low | 12 | 116 | 128 |  | 4 | 36 | 40 | 3 | 9 | | 12 | |
| Medium | 51 | 207 | 256 |  | 30 | 90 | 120 | 15 | 111 | | 126 | |
| High | 15 | 19 | 34 |  | 2 | 17 | 19 | 10 | 2 | | 12 | |
| **Ca** |  |  |  | <0.001 |  |  |  |  |  | |  | |
| Low | 2 | 20 | 22 |  | 0 | 9 | 9 | 1 | 17 | | 18 | |
| Medium | 60 | 307 | 367 |  | 32 | 123 | 155 | 21 | 98 | | 119 | |
| High | 16 | 15 | 31 |  | 4 | 11 | 15 | 26 | 7 | | 13 | |
| **P** |  |  |  | 0.543 |  |  |  |  |  | |  | |
| Low | 3 | 6 | 9 |  | 0 | 7 | 7 | 2 | 5 | | 7 | |
| Medium | 72 | 320 | 392 |  | 36 | 133 | 169 | 22 | 107 | | 129 | |
| High | 3 | 16 | 19 |  | 0 | 3 | 3 | 4 | 10 | | 14 | |

Table S2. Distribution of variables according to osteoporosis status

| **Factors** | **Univariate Analysis** | **Multivariate Logistic Analysis** | | |
| --- | --- | --- | --- | --- |
|  | **P** | **OR** | **95%CI** | **P** |
| **Age** | <0.001*** | 1.112 | 1.066-1.666 | <0.001*** |
| **BMI** | <0.001*** |  |  |  |
| <18.5 |  | - | - | - |
| 18.5-27.99 |  | 10.125 | 2.026-54.027 | 0.005269** |
| ≥28 |  | 0.547 | 0.085-2.418 | 0.472181 |
| **Smoke** | <0.001*** |  |  |  |
| No |  | - | - | - |
| Yes |  | 11.124 | 0.801-333.865 | 0.095779 |
| **Alcohol** | <0.001*** |  |  |  |
| No |  | - | - | - |
| Yes |  | 3.387 | 0.735-14.264 | 0.101140 |
| **M stage** | <0.001*** |  |  |  |
| 0 |  | - | - | - |
| 1 |  | 1.806 | 0.528-5.704 | 0.324586 |
| **Molecular type** | <0.001*** |  |  |  |
| HER+/HR- |  | - | - | - |
| HER+/HR+ |  | 0.023 | 0.002-0.128 | <0.001*** |
| Triple-negtive |  | 1.863 | 0.427-7.694 | 0.390087 |
| LuminalA |  | 0.029 | 0.003-0.198 | 0.001158** |
| LuminalB |  | 0.032 | 0.003-0.222 | 0.001482** |
| **Surgery** | 0.986 |  |  |  |
| No |  |  |  |  |
| Yes |  |  |  |  |
| **Anti-estrogens** | <0.001*** |  |  |  |
| No |  | - | - | - |
| Aromatase inhibitor |  | 121.986 | 22.247-1089.162 | <0.001*** |
| Anti-Estrogen R |  | 55.718 | 4.358-808.797 | 0.001956** |
| Combination |  | 105.181 | 2.380-4381.598 | 0.018562* |
| **Chemotherapy** | <0.001*** |  |  |  |
| No |  | - | - | - |
| Yes |  | 3.337 | 1.249-9.623 | 0.019915* |
| **Targeted therapy** | <0.001*** |  |  |  |
| No |  | - |  |  |
| Yes |  | 0.829 | 0.266-2.325 | 0.731836 |
| **GC** | <0.001*** |  |  |  |
| No |  | - | - | - |
| Yes |  | 4.261 | 1.884-10.336 | 0.001110** |
| **Radiotherapy** | <0.001*** |  |  |  |
| No |  | - | - | - |
| Yes |  | 0.769 | 0.320-1.766 | 0.544425 |
| **Family history** | <0.001*** |  |  |  |
| No |  | - | - | - |
| Yes |  | 15.543 | 1.963-207.712 | 0.017321* |
| **History of fracture** | <0.001*** |  |  |  |
| No |  | - | - | - |
| Yes |  | 5.060 | 1.669-15.465 | 0.004051** |
| **Karnofskybelow40** | <0.001*** |  |  |  |
| No |  | - | - | - |
| Yes |  | 2.266 | 0.374-12.930 | 0.362312 |
| **BALP** | <0.001*** |  |  |  |
| Low |  | 0.327 | 0.106-0.909 | 0.039364* |
| Medium |  | - | - | - |
| High |  | 4.840 | 1.519-15.864 | 0.007878** |
| **Ca** | <0.001*** |  |  |  |
| Low |  | 0.429 | 0.023-2.976 | 0.467712 |
| Medium |  | - | - | - |
| High |  | 3.410 | 0.939-12.233 | 0.059657 |
| **P** | <0.001*** |  |  |  |
| Low |  | 0.176 | 0.001-10.679 | 0.522127 |
| Medium |  | - | - | - |
| High |  | 0.530 | 0.051-2.994 | 0.529769 |

Table S3. Univariate and multivariate logistic analyses of osteoporosis in breast cancer patients.

|  | **Training**  **group**  **(n=420)** | **%** | **Internal**  **Validation group**  **(n=179)** | **%** | **χ^2^** | **P** | **External validation group**  **(n=150)** | **%** |
| --- | --- | --- | --- | --- | --- | --- | --- | --- |
| **BMI** |  |  |  |  | 1.205 | 0.548 |  |  |
| <18.5 | 17 | 4.0 | 9 | 84.9 |  |  | 2 | 1.3 |
| 18.5-27.99 | 373 | 88.0 | 152 | 5.0 |  |  | 142 | 94.6 |
| ≥28 | 33 | 7.8 | 18 | 10.0 |  |  | 6 | 4.0 |
| **Menopause** |  |  |  |  | 6.811 | 0.009 |  |  |
| No | 189 | 45.0 | 60 | 33.5 |  |  | 72 | 48.0 |
| Yes | 231 | 55.0 | 119 | 66.4 |  |  | 78 | 52.0 |
| **Smoke** |  |  |  |  | 0.225 | 0.636 |  |  |
| No | 413 | 98.3 | 175 | 97.7 |  |  | 147 | 98.0 |
| Yes | 7 | 1.6 | 4 | 2.2 |  |  | 3 | 2.0 |
| **Alcohol** |  |  |  |  | 0.344 | 0.563 |  |  |
| No | 399 | 95.0 | 172 | 96.0 |  |  | 145 | 96.6 |
| Yes | 21 | 5.0 | 7 | 3.9 |  |  | 5 | 3.3 |
| **M stage** |  |  |  |  | 0.985 | 0.328 |  |  |
| 0 | 369 | 87.8 | 152 | 84.9 |  |  | 128 | 85.3 |
| 1 | 51 | 12.1 | 27 | 15.0 |  |  | 22 | 14.6 |
| **Molecular type** |  |  |  |  | 3.243 | 0.518 |  |  |
| HER+/HR- | 103 | 24.5 | 38 | 21.2 |  |  | 22 | 14.6 |
| HER+/HR+ | 163 | 38.8 | 82 | 45.8 |  |  | 71 | 47.3 |
| Triple-negtive | 45 | 10.7 | 17 | 9.4 |  |  | 14 | 9.3 |
| LuminalA | 54 | 12.8 | 24 | 13.4 |  |  | 28 | 18.6 |
| LuminalB | 55 | 13.0 | 18 | 10.0 |  |  | 15 | 10.0 |
| **Surgery** |  |  |  |  | 0 | 0.290 |  |  |
| No | 7 | 1.6 | 3 | 1.6 |  |  | 9 | 6.0 |
| Yes | 413 | 98.3 | 176 | 98.3 |  |  | 141 | 94.0 |
| **Anti-estrogens** |  |  |  |  | 1.354 | 0.716 |  |  |
| No | 198 | 47.1 | 78 | 42.4 |  |  | 64 | 42.6 |
| Aromataseinhibitor | 182 | 43.3 | 87 | 48.6 |  |  | 54 | 36.0 |
| Anti-estrogenR | 33 | 7.8 | 14 | 7.8 |  |  | 30 | 20.0 |
| Combination | 7 | 1.6 | 2 | 1.1 |  |  | 2 | 1.3 |
| **Chemotherapy** |  |  |  |  | 1.933 | 0.164 |  |  |
| No | 117 | 27.8 | 60 | 33.5 |  |  | 27 | 18.0 |
| Yes | 303 | 78.1 | 119 | 66.4 |  |  | 123 | 82.0 |
| **Targeted therapy** |  |  |  |  | 3.260 | 0.071 |  |  |
| No | 346 | 82.3 | 158 | 88.2 |  |  | 118 | 78.6 |
| Yes | 74 | 17.6 | 21 | 11.7 |  |  | 32 | 21.3 |
| **GC** |  |  |  |  | 1.730 | 0.188 |  |  |
| No | 210 | 50.0 | 79 | 44.1 |  |  | 36 | 24.0 |
| Yes | 210 | 50.0 | 100 | 55.8 |  |  | 114 | 76.0 |
| **Radiotherapy** |  |  |  |  | 1.965 | 0.161 |  |  |
| No | 323 | 76.9 | 128 | 71.5 |  |  | 116 | 77.3 |
| Yes | 97 | 23.0 | 51 | 28.4 |  |  | 34 | 22.6 |
| **Family history** |  |  |  |  | 0.383 | 0.536 |  |  |
| No | 404 | 96.1 | 174 | 97.2 |  |  | 145 | 96.6 |
| Yes | 16 | 3.8 | 5 | 2.7 |  |  | 5 | 3.3 |
| **History of fracture** |  |  |  |  | 0.461 | 0.497 |  |  |
| No | 385 | 91.6 | 167 | 93.2 |  |  | 121 | 80.6 |
| Yes | 35 | 8.3 | 12 | 6.7 |  |  | 29 | 19.3 |
| **Karnofskybelow40** |  |  |  |  | 0.044 | 0.834 |  |  |
| No | 402 | 95.7 | 172 | 96.0 |  |  | 144 | 96.0 |
| Yes | 18 | 4.2 | 7 | 3.9 |  |  | 6 | 4.0 |
| **BALP** |  |  |  |  | 1.489 | 0.457 |  |  |
| Low | 125 | 29.0 | 46 | 25.6 |  |  | 12 | 8.0 |
| Medium | 259 | 61.6 | 119 | 66.4 |  |  | 126 | 84.0 |
| High | 39 | 9.2 | 14 | 7.8 |  |  | 12 | 8.0 |
| **Ca** |  |  |  |  | 0.903 | 0.637 |  |  |
| Low | 21 | 5.0 | 10 | 5.5 |  |  | 18 | 12.0 |
| Medium | 364 | 86.6 | 158 | 88.2 |  |  | 119 | 79.3 |
| High | 35 | 8.3 | 11 | 6.1 |  |  | 13 | 8.6 |
| **P** |  |  |  |  | 3.322 | 0.200 |  |  |
| Low | 8 | 1.9 | 8 | 4.4 |  |  | 7 | 4.6 |
| Medium | 396 | 94.2 | 165 | 92.1 |  |  | 129 | 86.0 |
| High | 16 | 3.8 | 6 | 3.3 |  |  | 14 | 9.3 |
| **Osteoporosis** |  |  |  |  | 0.059 | 0.808 |  |  |
| No | 339 | 80.7 | 146 | 81.5 |  |  | 122 | 81.3 |
| Yes | 81 | 19.2 | 33 | 18.4 |  |  | 28 | 18.6 |

Table S4.Baseline clinical characteristics of breast cancer patients in fracture model.( We used t-test to verify the randomness of the age factor,p=0.597 (95%CI,-4.835--0.971))

|  | **Training Cohort** | | | | **Internal Validation Cohort** | | | **External Validation Cohort** | | | | |
| --- | --- | --- | --- | --- | --- | --- | --- | --- | --- | --- | --- | --- |
|  | **Fracture** | **Normal** | **Total** |  | **Fracture** | **Normal** | **Total** | **Fracture** | | **Normal** | | **Total** |
| **Variable** | N=26 | N=394 | N=420 | P | N=15 | N=164 | N=179 | N=12 | N=138 | | N=150 | |
| **Age** | - | - | - | - | - | - | - | - | - | | - | |
| BMI |  |  |  | 0.056 |  |  |  |  |  | |  | |
| <18.5 | 4 | 13 | 17 |  | 5 | 4 | 9 | 1 | 1 | | 2 | |
| 18.5-27.99 | 20 | 350 | 370 |  | 8 | 144 | 152 | 11 | 132 | | 143 | |
| ≥28 | 2 | 31 | 33 |  | 2 | 16 | 18 | 0 | 6 | | 6 | |
| **Menopause** |  |  |  | 0.003 |  |  |  |  |  | |  | |
| No | 4 | 185 | 189 |  | 0 | 60 | 60 | 0 | 72 | | 72 | |
| Yes | 22 | 209 | 231 |  | 15 | 104 | 119 | 12 | 66 | | 78 | |
| **Smoke** |  |  |  | <0.001*** |  |  |  |  |  | |  | |
| No | 23 | 390 | 413 |  | 14 | 161 | 175 | 12 | 135 | | 147 | |
| Yes | 3 | 4 | 7 |  | 1 | 3 | 4 | 0 | 3 | | 3 | |
| **Alcohol** |  |  |  | 0.041 |  |  |  |  |  | |  | |
| No | 22 | 377 | 398 |  | 14 | 158 | 172 | 12 | 133 | | 145 | |
| Yes | 4 | 17 | 21 |  | 1 | 6 | 7 | 0 | 5 | | 5 | |
| **M stage** |  |  |  | 0.078 |  |  |  |  |  | |  | |
| 0 | 20 | 349 | 367 |  | 12 | 140 | 152 | 12 | 116 | | 128 | |
| 1 | 6 | 45 | 51 |  | 3 | 24 | 27 | 0 | 22 | | 22 | |
| **Molecular type** |  |  |  | 0.047 |  |  |  |  |  | |  | |
| HER+/HR- | 2 | 101 | 103 |  | 1 | 37 | 38 | 0 | 22 | | 22 | |
| HER+/HR+ | 15 | 148 | 163 |  | 8 | 74 | 82 | 4 | 67 | | 71 | |
| Triple-negtive | 1 | 44 | 45 |  | 2 | 15 | 17 | 2 | 12 | | 14 | |
| LuminalA | 2 | 52 | 54 |  | 1 | 23 | 24 | 3 | 25 | | 28 | |
| LuminalB | 6 | 49 | 55 |  | 3 | 15 | 18 | 3 | 12 | | 15 | |
| **Surgery** |  |  |  | 1 |  |  |  |  |  | |  | |
| No | 0 | 7 | 7 |  | 0 | 3 | 3 | 12 | 9 | | 21 | |
| Yes | 26 | 387 | 413 |  | 15 | 161 | 176 | 0 | 129 | | 129 | |
| **Anti-estrogens** |  |  |  | <0.001*** |  |  |  |  |  | |  | |
| No | 1 | 197 | 198 |  | 2 | 74 | 76 | 5 | 59 | | 64 | |
| Aromataseinhibitor | 24 | 158 | 182 |  | 13 | 74 | 87 | 7 | 47 | | 54 | |
| Anti-estrogenR | 0 | 33 | 33 |  | 0 | 14 | 14 | 0 | 30 | | 30 | |
| Combination | 1 | 6 | 7 |  | 0 | 2 | 2 | 0 | 2 | | 2 | |
| **Chemotherapy** |  |  |  | 0.311 |  |  |  |  |  | |  | |
| No | 5 | 112 | 120 |  | 2 | 58 | 60 | 2 | 25 | | 27 | |
| Yes | 21 | 282 | 300 |  | 13 | 106 | 119 | 10 | 113 | | 123 | |
| **Targeted therapy** |  |  |  | 0.362 |  |  |  |  |  | |  | |
| No | 24 | 329 | 353 |  | 12 | 136 | 148 | 20 | 109 | | 129 | |
| Yes | 2 | 65 | 67 |  | 3 | 28 | 31 | 8 | 29 | | 37 | |
| **GC** |  |  |  | 0.686 |  |  |  |  |  | |  | |
| No | 12 | 198 | 210 |  | 3 | 76 | 79 | 9 | 33 | | 42 | |
| Yes | 14 | 196 | 210 |  | 12 | 88 | 100 | 3 | 105 | | 108 | |
| **Radiotherapy** |  |  |  | 0.998 |  |  |  |  |  | |  | |
| No | 20 | 303 | 323 |  | 13 | 115 | 128 | 10 | 106 | | 116 | |
| Yes | 6 | 91 | 97 |  | 2 | 49 | 51 | 2 | 32 | | 34 | |
| **Family history** |  |  |  | 0.110 |  |  |  |  |  | |  | |
| No | 23 | 381 | 404 |  | 12 | 162 | 174 | 9 | 136 | | 145 | |
| Yes | 3 | 13 | 16 |  | 3 | 2 | 5 | 3 | 2 | | 5 | |
| **History of fracture** |  |  |  | <0.001*** |  |  |  |  |  | |  | |
| No | 19 | 366 | 385 |  | 11 | 156 | 167 | 7 | 114 | | 121 | |
| Yes | 7 | 28 | 35 |  | 4 | 8 | 12 | 5 | 24 | | 29 | |
| **Karnofsky below40** |  |  |  | 0.166 |  |  |  |  |  | |  | |
| No | 23 | 379 | 402 |  | 13 | 159 | 172 | 11 | 133 | | 144 | |
| Yes | 3 | 15 | 18 |  | 2 | 5 | 7 | 1 | 5 | | 6 | |
| **BALP** |  |  |  | 0.002** |  |  |  |  |  | |  | |
| Low | 3 | 119 | 122 |  | 1 | 108 | 109 | 0 | 12 | | 12 | |
| Medium | 15 | 244 | 259 |  | 11 | 45 | 56 | 6 | 120 | | 126 | |
| High | 8 | 31 | 39 |  | 3 | 11 | 14 | 8 | 6 | | 14 | |
| **Ca** |  |  |  | 0.006** |  |  |  |  |  | |  | |
| Low | 0 | 21 | 21 |  | 1 | 9 | 10 | 1 | 17 | | 18 | |
| Medium | 19 | 345 | 364 |  | 13 | 145 | 158 | 7 | 112 | | 119 | |
| High | 7 | 28 | 35 |  | 1 | 10 | 11 | 4 | 9 | | 13 | |
| **P** |  |  |  | 0.783 |  |  |  |  |  | |  | |
| Low | 0 | 9 | 8 |  | 1 | 7 | 8 | 1 | 6 | | 7 | |
| Medium | 26 | 370 | 396 |  | 13 | 152 | 165 | 8 | 121 | | 129 | |
| High | 0 | 16 | 16 |  | 1 | 5 | 6 | 3 | 11 | | 14 | |
| **Osteoporosis** |  |  |  | <0.001*** |  |  |  |  |  | |  | |
| No | 6 | 333 | 339 |  | 3 | 143 | 146 | 4 | 118 | | 122 | |
| Yes | 26 | 61 | 81 |  | 12 | 21 | 33 | 8 | 20 | | 28 | |

Table S5. Distribution of variables according to fracture status

| **Factors** | **Univariate Analysis** | **Multivariate Logistic Analysis** | | |
| --- | --- | --- | --- | --- |
|  | **P** | **OR** | **95%CI** | **P** |
| **Age** | <0.001*** | 1.241 | 1.118- 1.413 | <0.001 *** |
| **BMI** | <0.001*** |  |  |  |
| <18.5 |  | - | - | - |
| 18.5-27.99 |  | 32.247 | 3.290-469.268 | 0.004737 ** |
| ≥28 |  | 1.104 | 0.088-9.834 | 0.932135 |
| **Menopause** | <0.001*** |  |  |  |
| No |  | - | - | - |
| Yes |  | 0.128 | 0.010-1.340 | 0.089392 |
| **Smoke** | <0.001*** |  |  |  |
| No |  | - | - | - |
| Yes |  | 3.063 | 0.057-304.117 | 0.596344 |
| **Alcohol** | <0.001*** |  |  |  |
| No |  | - | - | - |
| Yes |  | 0.994 | 0.033-12.615 | 0.997178 |
| **M stage** | <0.001*** |  |  |  |
| 0 |  | - | - | - |
| 1 |  | 6.589 | 0.959-58.011 | 0.064569 |
| **Molecular type** | <0.001*** |  |  |  |
| HER+/HR- |  | - | - | - |
| HER+/HR+ |  | 1.711 | 0.113-40.588 | 0.713240 |
| Triple-negtive |  | 2.703588e+07 | 1.659747e-33-NA | 0.991586 |
| LuminalA |  | 0.058 | 0.001- 2.107 | 0.131609 |
| LuminalB |  | 5.120 | 0.365-107.688 | 0.249701 |
| **Surgery** | 0.988 |  |  |  |
| No |  |  |  |  |
| Yes |  |  |  |  |
| **Anti-estrogens** | <0.001*** |  |  |  |
| No |  | - | - | - |
| Aromatase inhibitor |  | 1.797799e+09 | 5.815862e-31-NA | 0.989522 |
| Anti-Estrogen R |  | 102.050 | 1.77e-45-1.57e+47 | 0.999141 |
| Combination |  | 5.894215e+09 | 1.906655e-30-NA | 0.988938 |
| **Chemotherapy** | <0.001*** |  |  |  |
| No |  | - | - | - |
| Yes |  | 39.539 | 4.951-580.162 | 0.002024 ** |
| **Targeted therapy** | <0.001*** |  |  |  |
| No |  | - |  |  |
| Yes |  | 0.087 | 0.114334 | 0.114334 |
| **GC** | <0.001*** |  |  |  |
| No |  | - | - | - |
| Yes |  | 1.401 | 0.305-6.916 | 0.663943 |
| **Radiotherapy** | <0.001*** |  |  |  |
| No |  | - | - | - |
| Yes |  | 3.785 | 0.620-25.697 | 0.149754 |
| **Family history** | <0.001*** |  |  |  |
| No |  | - | - | - |
| Yes |  | 6.690 | 0.422-114.353 | 0.175526 |
| **History of fracture** | <0.001*** |  |  |  |
| No |  | - | - | - |
| Yes |  | 9.484 | 1.504-82.176 | 0.023964 * |
| **Karnofskybelow40** | <0.001*** |  |  |  |
| No |  | - | - | - |
| Yes |  | 0.386 | 0.033- 3.060 | 0.397657 |
| **BALP** | <0.001*** |  |  |  |
| Low |  | 1.098 | 0.085-10.433 | 0.937110 |
| Medium |  | - | - | - |
| High |  | 59.933 | 7.245-836.355 | <0.001 *** |
| **Ca** | <0.001*** |  |  |  |
| Low |  | 2.167947e-07 | NA- 2.1812e+109 | 0.997385 |
| Medium |  | - | - | - |
| High |  | 1.530 | 0.258-8.715 | 0.628808 |
| **P** | <0.001*** |  |  |  |
| Low |  | 8.998370e-09 | NA-3.8319e+207 | 0.998268 |
| Medium |  | - | - | - |
| High |  | 1.109471e-08 | NA-6.6637e+118 | 0.997287 |
| **Osteoporosis** | <0.001*** |  |  |  |
| No |  | - | - | - |
| Yes |  | 6.457 | 1.284-39.443 | 0.029193 * |

Table S6. Univariate and multivariate logistic analyses of fracture in breast cancer patients.

|  | **Training**  **group**  **(n=420)** | **%** | **Internal**  **Validation group**  **(n=179)** | **%** | **χ^2^** | **P** | **External validation group**  **(n=150)** | **%** |
| --- | --- | --- | --- | --- | --- | --- | --- | --- |
| **Menopause** |  |  |  |  | 1.025 | 0.311 |  |  |
| No | 169 | 40.2 | 80 | 44.6 |  |  | 72 | 48.0 |
| Yes | 251 | 59.7 | 99 | 55.3 |  |  | 78 | 52.0 |
| **Smoke** |  |  |  |  | 0.047 | 0.481 |  |  |
| No | 414 | 98.5 | 175 | 97.7 |  |  | 147 | 98.0 |
| Yes | 6 | 1.4 | 4 | 2.2 |  |  | 3 | 2.0 |
| **Alcohol** |  |  |  |  | 0.477 | 0.490 |  |  |
| No | 402 | 95..7 | 169 | 94.4 |  |  | 145 | 96.6 |
| Yes | 28 | 4.2 | 10 | 5.5 |  |  | 5 | 3.3 |
| **T stage** |  |  |  |  | 2.991 | 0.373 |  |  |
| T0/Tis | 2 | 0.4 | 0 | 0 |  |  | 2 | 1.3 |
| T1 | 220 | 52.5 | 106 | 59.2 |  |  | 63 | 42.2 |
| T2 | 161 | 38.3 | 57 | 31.8 |  |  | 71 | 47.3 |
| T3 | 37 | 8.8 | 16 | 8.9 |  |  | 14 | 9.3 |
| **N stage** |  |  |  |  | 2.085 | 0.755 |  |  |
| N0 | 273 | 65.0 | 124 | 69.2 |  |  | 99 | 66.0 |
| N1 | 84 | 20.0 | 32 | 17.8 |  |  | 35 | 23.0 |
| N2 | 45 | 10.7 | 19 | 10.6 |  |  | 10 | 6.6 |
| N3 | 17 | 4.0 | 4 | 2.2 |  |  | 6 | 4.0 |
| N4 | 1 | 0.2 | 0 | 0 |  |  | 0 | 0 |
| **Molecular type** |  |  |  |  | 0.418 | 0.077 |  |  |
| HER+/HR- | 98 | 23.3 | 43 | 24.0 |  |  | 22 | 14.6 |
| HER+/HR+ | 179 | 42.6 | 66 | 36.8 |  |  | 71 | 47.3 |
| Triple-negtive | 46 | 10.9 | 16 | 8.9 |  |  | 14 | 9.3 |
| LuminalA | 56 | 13.3 | 22 | 12.2 |  |  | 28 | 18.6 |
| LuminalB | 41 | 9.7 | 32 | 17.8 |  |  | 15 | 10.0 |
| **Surgery** |  |  |  |  | 0.116 | 0.734 |  |  |
| No | 8 | 1.9 | 2 | 1.1 |  |  | 9 | 6.0 |
| Yes | 412 | 98.0 | 177 | 98.8 |  |  | 141 | 94.0 |
| **Anti-estrogens** |  |  |  |  | 0.580 | 0.466 |  |  |
| No | 190 | 45.2 | 84 | 46.9 |  |  | 64 | 42.6 |
| Yes | 230 | 54.7 | 95 | 53.0 |  |  | 54 | 36.0 |
| **Chemotherapy** |  |  |  |  | 0.580 | 0.446 |  |  |
| No | 128 | 30.4 | 49 | 46.9 |  |  | 27 | 18.0 |
| Yes | 292 | 69.5 | 130 | 53.0 |  |  | 123 | 82.0 |
| **Targeted therapy** |  |  |  |  | 8.049 | 0.005 |  |  |
| No | 365 | 86.0 | 139 | 77.6 |  |  | 118 | 78.6 |
| Yes | 55 | 13.0 | 40 | 22.3 |  |  | 32 | 21.3 |
| **Radiotherapy** |  |  |  |  | 0.765 | 0.382 |  |  |
| No | 312 | 74.2 | 139 | 77.6 |  |  | 116 | 77.3 |
| Yes | 108 | 25.7 | 40 | 22.3 |  |  | 34 | 22.6 |
| **Osteoporosis** |  |  |  |  | 0.059 | 0.808 |  |  |
| No | 339 | 80.7 | 146 | 81.5 |  |  | 122 | 81.3 |
| Yes | 81 | 19.2 | 33 | 18.4 |  |  | 28 | 13.6 |
| **Brain metastasis** |  |  |  |  | 0.601 | 0.408 |  |  |
| No | 400 | 95.2 | 173 | 96.6 |  |  | 146 | 97.3 |
| Yes | 20 | 4.7 | 6 | 3.3 |  |  | 4 | 2.6 |
| **Liver metastasis** |  |  |  |  | 6.370 | 0.012 |  |  |
| No | 392 | 93.3 | 176 | 98.3 |  |  | 143 | 95.3 |
| Yes | 28 | 6.6 | 3 | 1.6 |  |  | 7 | 4.6 |
| **Lung metastasis** |  |  |  |  | 0.540 | 0.462 |  |  |
| No | 387 | 92.1 | 168 | 93.8 |  |  | 137 | 91.3 |
| Yes | 33 | 7.8 | 11 | 6.1 |  |  | 13 | 8.6 |

Table S7.Baseline clinical characteristics of breast cancer patients in prgnosis model.( We used t-test to verify the randomness of the age factor,p=0.597 (95%CI,-0.379-1.512))

|  | **Training Cohort** | | | | **Internal Validation Cohort** | | | **External Validation Cohort** | | | | |
| --- | --- | --- | --- | --- | --- | --- | --- | --- | --- | --- | --- | --- |
|  | **Dead** | **Alive** | **Total** |  | **Dead** | **Alive** | **Total** | **Dead** | | **Alive** | | **Total** |
| **Variable** | N=104 | N=316 | N=420 | P | N=47 | N=132 | N=179 | N=29 | N=121 | | N=150 | |
| **Age** | - | - | - | - | - | - | - | - | - | | - | |
| **Menopause** |  |  |  | 0.264 |  |  |  |  |  | |  | |
| No | 37 | 132 | 169 |  | 15 | 65 | 80 | 14 | 58 | | 72 | |
| Yes | 67 | 184 | 251 |  | 32 | 67 | 99 | 15 | 63 | | 78 | |
| **Smoke** |  |  |  | 0.334 |  |  |  |  |  | |  | |
| No | 101 | 313 | 414 |  | 44 | 131 | 175 | 29 | 118 | | 147 | |
| Yes | 3 | 3 | 6 |  | 3 | 7 | 10 | 0 | 3 | | 3 | |
| **Alcohol** |  |  |  | 0.156 |  |  |  |  |  | |  | |
| No | 97 | 305 | 402 |  | 44 | 125 | 169 | 29 | 116 | | 145 | |
| Yes | 7 | 11 | 18 |  | 3 | 7 | 10 | 0 | 3 | | 3 | |
| **T stage** |  |  |  | <0.001*** |  |  |  |  |  | |  | |
| T0 | 0 | 2 | 2 |  | 0 | 0 | 0 | 0 | 0 | | 0 | |
| T1 | 24 | 196 | 220 |  | 15 | 91 | 106 | 7 | 56 | | 63 | |
| T2 | 56 | 105 | 161 |  | 22 | 35 | 57 | 15 | 56 | | 71 | |
| T3 | 24 | 13 | 37 |  | 10 | 6 | 16 | 6 | 8 | | 14 | |
| T4 | 0 | 0 | 0 |  | 0 | 0 | 0 | 1 | 1 | | 2 | |
| **N stage** |  |  |  | <0.001*** |  |  |  |  |  | |  | |
| N0 | 28 | 245 | 273 |  | 14 | 110 | 124 | 7 | 92 | | 99 | |
| N1 | 30 | 54 | 84 |  | 13 | 19 | 32 | 10 | 25 | | 35 | |
| N2 | 29 | 16 | 45 |  | 16 | 3 | 19 | 8 | 2 | | 10 | |
| N3 | 16 | 1 | 17 |  | 4 | 0 | 4 | 4 | 2 | | 6 | |
| N4 | 1 | 0 | 1 |  | 0 | 0 | 0 | 0 | 0 | | 0 | |
| **Molecular type** |  |  |  | <0.001*** |  |  |  |  |  | |  | |
| HER+/HR- | 13 | 85 | 98 |  | 8 | 35 | 43 | 0 | 16 | | 16 | |
| HER+/HR+ | 36 | 143 | 179 |  | 17 | 49 | 66 | 4 | 63 | | 67 | |
| Triple-negtive | 27 | 19 | 46 |  | 11 | 5 | 16 | 2 | 7 | | 9 | |
| LuminalA | 10 | 46 | 56 |  | 2 | 20 | 22 | 3 | 26 | | 29 | |
| LuminalB | 18 | 26 | 44 |  | 9 | 23 | 32 | 3 | 9 | | 12 | |
| **Surgery** |  |  |  | 0.037 |  |  |  |  |  | |  | |
| No | 5 | 3 | 8 |  | 2 | 0 | 2 | 12 | 4 | | 16 | |
| Yes | 99 | 313 | 412 |  | 45 | 132 | 177 | 0 | 117 | | 117 | |
| **Anti-estrogens** |  |  |  | 0.502 |  |  |  |  |  | |  | |
| No | 50 | 140 | 190 |  | 24 | 60 | 84 | 5 | 49 | | 54 | |
| Yes | 54 | 176 | 230 |  | 23 | 72 | 95 | 7 | 72 | | 79 | |
| **Chemotherapy** |  |  |  | 0.247 |  |  |  |  |  | |  | |
| No | 38 | 99 | 137 |  | 20 | 29 | 49 | 2 | 25 | | 27 | |
| Yes | 66 | 226 | 292 |  | 27 | 103 | 130 | 10 | 96 | | 106 | |
| **Targeted therapy** |  |  |  | 0.836 |  |  |  |  |  | |  | |
| No | 91 | 274 | 365 |  | 38 | 101 | 139 | 20 | 97 | | 117 | |
| Yes | 13 | 42 | 55 |  | 9 | 31 | 40 | 8 | 24 | | 32 | |
| **Radiotherapy** |  |  |  | 0.061 |  |  |  |  |  | |  | |
| No | 70 | 242 | 312 |  | 35 | 104 | 139 | 10 | 92 | | 102 | |
| Yes | 34 | 74 | 108 |  | 12 | 28 | 40 | 2 | 29 | | 31 | |
| **Osteoporosis** |  |  |  | <0.001*** |  |  |  |  |  | |  | |
| No | 72 | 271 | 343 |  | 34 | 108 | 142 | 4 | 107 | | 111 | |
| Yes | 32 | 45 | 77 |  | 13 | 24 | 37 | 8 | 14 | | 22 | |
| **Brain metastasis** |  |  |  | <0.001*** |  |  |  |  |  | |  | |
| No | 84 | 316 | 400 |  | 41 | 132 | 173 | 25 | 121 | | 146 | |
| Yes | 20 | 0 | 20 |  | 6 | 0 | 6 | 4 | 0 | | 4 | |
| **Liver metastasis** |  |  |  | <0.001*** |  |  |  |  |  | |  | |
| No | 76 | 316 | 392 |  | 44 | 132 | 176 | 23 | 120 | | 143 | |
| Yes | 28 | 0 | 28 |  | 3 | 0 | 3 | 6 | 1 | | 7 | |
| **Lung metastasis** |  |  |  | <0.001*** |  |  |  |  |  | |  | |
| No | 71 | 316 | 387 |  | 36 | 132 | 168 | 19 | 118 | | 137 | |
| Yes | 33 | 0 | 33 |  | 11 | 0 | 11 | 10 | 3 | | 13 | |

Table S8. Distribution of variables according to prognosis status

| **Factors** | **Univariate Analysis** | **Multivariate Logistic Analysis** | | |
| --- | --- | --- | --- | --- |
|  | **P** | **OR** | **95%CI** | **P** |
| **Age** | <0.001*** | 1.241 | 1.118- 1.413 | <0.001 *** |
| **BMI** | <0.001*** |  |  |  |
| <18.5 |  | 32.247 | 3.290-469.268 | 0.004737 ** |
| 18.5-27.99 |  | - | - | - |
| ≥28 |  | 1.104 | 0.088-9.834 | 0.932135 |
| **Smoke** | <0.001*** |  |  |  |
| No |  | - | - | - |
| Yes |  | 3.063 | 0.057-304.117 | 0.596344 |
| **Alcohol** | <0.001*** |  |  |  |
| No |  | - | - | - |
| Yes |  | 0.994 | 0.033-12.615 | 0.997178 |
| **M stage** | <0.001*** |  |  |  |
| 0 |  | - | - | - |
| 1 |  | 6.589 | 0.959-58.011 | 0.064569 |
| **Molecular type** | <0.001*** |  |  |  |
| HER+/HR- |  | - | - | - |
| HER+/HR+ |  | 1.711 | 0.113-40.588 | 0.713240 |
| Triple-negtive |  | 2.703588e+07 | 1.659747e-33-NA | 0.991586 |
| LuminalA |  | 0.058 | 0.001- 2.107 | 0.131609 |
| LuminalB |  | 5.120 | 0.365-107.688 | 0.249701 |
| **Surgery** | 0.988 |  |  |  |
| No |  |  |  |  |
| Yes |  |  |  |  |
| **Anti-strogens** | <0.001*** |  |  |  |
| No |  | - | - | - |
| Aromatase inhibitor |  | 1.797799e+09 | 5.815862e-31-NA | 0.989522 |
| Anti-Estrogen R |  | 102.050 | 1.77e-45-1.57e+47 | 0.999141 |
| Combination |  | 5.894215e+09 | 1.906655e-30-NA | 0.988938 |
| **Chemotherapy** | <0.001*** |  |  |  |
| No |  | - | - | - |
| Yes |  | 39.539 | 4.951-580.162 | 0.002024 ** |
| **Targeted therapy** | <0.001*** |  |  |  |
| No |  | - |  |  |
| Yes |  | 0.087 | 0.114334 | 0.114334 |
| **GC** | <0.001*** |  |  |  |
| No |  | - | - | - |
| Yes |  | 1.401 | 0.305-6.916 | 0.663943 |
| **Radiotherapy** | <0.001*** |  |  |  |
| No |  | - | - | - |
| Yes |  | 3.785 | 0.620-25.697 | 0.149754 |
| **Family history** | <0.001*** |  |  |  |
| No |  | - | - | - |
| Yes |  | 6.690 | 0.422-114.353 | 0.175526 |
| **History of fracture** | <0.001*** |  |  |  |
| No |  | - | - | - |
| Yes |  | 9.484 | 1.504-82.176 | 0.023964 * |
| **Karnofskybelow40** | <0.001*** |  |  |  |
| No |  | - | - | - |
| Yes |  | 0.386 | 0.033- 3.060 | 0.397657 |
| **BALP** | <0.001*** |  |  |  |
| Low |  | 1.098 | 0.085-10.433 | 0.937110 |
| Medium |  | - | - | - |
| High |  | 59.933 | 7.245-836.355 | <0.01 *** |
| **Ca** | <0.001*** |  |  |  |
| Low |  | 2.167947e-07 | NA- 2.1812e+109 | 0.997385 |
| Medium |  | - | - | - |
| High |  | 1.530 | 0.258-8.715 | 0.628808 |
| **P** |  |  |  |  |
| Low | 0.267 | 8.998370e-09 | NA-3.8319e+207 | 0.998268 |
| Medium | - | - | - | - |
| High | 0.777 | 1.109471e-08 | NA-6.6637e+118 | 0.997287 |
| **Osteoporosis** | <0.001*** |  |  |  |
| No |  | - | - | - |
| Yes |  | 6.457 | 1.284-39.443 | 0.029193 * |

Table S9. Univariate and multivariate logistic analyses of prognosis in breast cancer patients.
